# Mitochondrial dysfunction-induced high hCG associated with development of fetal growth restriction and pre-eclampsia with fetal growth restriction

**DOI:** 10.1038/s41598-022-07893-y

**Published:** 2022-03-08

**Authors:** Ryo Kiyokoba, Takeshi Uchiumi, Mikako Yagi, Takahiro Toshima, Shigehiro Tsukahara, Yasuyuki Fujita, Kiyoko Kato, Dongchon Kang

**Affiliations:** 1 Department of Clinical Chemistry and Laboratory Medicine, Graduate School of Medical Sciences, Kyushu University, Maidashi 3-1-1, Higashi-ku, Fukuoka, 812-8582 Japan; 2 Department of Obstetrics and Gynecology, Graduate School of Medical Sciences, Kyushu University, Maidashi 3-1-1, Higashi-ku, Fukuoka, 812-8582 Japan; 3 Department of Health Sciences, Graduate School of Medical Sciences, Kyushu University, Maidashi 3-1-1, Higashi-ku, Fukuoka, 812-8582 Japan

**Keywords:** Embryology, Translation

## Abstract

Fetal growth restriction (FGR) and pre-eclampsia with fetal growth restriction (PE/FGR) are high-risk perinatal diseases that may involve high levels of human chorionic gonadotropin (hCG) and mitochondrial dysfunction. However, little is known about how these factors affect placental function. We investigated how mitochondrial dysfunction and high hCG expression affected placental function in unexplained FGR and PE/FGR. We observed elevated expression of hCGβ and growth differentiation factor 15 mRNA and protein levels in the placenta with both diseases. Likewise, antiangiogenic factors, such as Ang2, IP10, sFlt1, IL8, IL1B, and TNFα, were also upregulated at the mRNA level. In addition, the expression of COXI and COXII which encoded by mitochondrial DNA were significantly decreased in both diseases, suggesting that mitochondrial translation was impaired. Treatment with hCG increased Ang2, IP10, IL8, and TNFα mRNA levels in a dose-dependent manner via the p38 and JNK pathways. Mitochondrial translation inhibitors increased hCGβ expression through stabilization of HIF1α, and increased IL8 and TNFα mRNA expression. These results revealed that high expression of hCG due to mitochondrial translational dysfunction plays an important role in the pathogenesis of FGR and PE/FGR.

## Introduction

Fetal growth restriction (FGR) is the failure of a fetus to achieve its genetic growth potential in utero and is associated with a significant risk factor of preterm birth, neonatal death, and stillbirth. It is a major cause of infant mortality and morbidity after preterm birth^1^. Pre-eclampsia (PE), which complicates about 3% of pregnancies, is characterized by hypertension and proteinuria occurring after 20th weeks of gestation or hypertension plus the involvement of at least one organ or system, and is associated with significant maternal and neonatal morbidity and mortality^2^.

Although the cause and pathogenesis of FGR and PE have not been fully defined, most cases are not associated with fetal congenital malformations, fetal genetic anomalies, or infectious etiology. The primary cause of FGR and PE has been reported to be ‘placental insufficiency’. This means that the fetus does not get enough nutrients and oxygen and is affected by a variety of factors, including changes in maternal or fetal blood flow, decreased oxygen, and poor adaptation to high oxygen and nutrients^3^. Placental ischemia due to impaired spiral artery remodeling and subsequent release of antiangiogenic factors has been considered important in the pathogenesis of these diseases^4,5^. Some unknown cases of FGR are etiologically related to PE because they show common pathological features in the placenta^6^.

Human chorionic gonadotropin (hCG) is an essential hormone for pregnancy and induces various physiological activities, such as maternal immune tolerance and the production of angiogenic factors^7^. hCG is produced by syncytiotrophoblasts (STB) and peaks between the 7th and 10th weeks of pregnancy, with a nadir at 18th weeks^8^. Several studies have demonstrated that patients with FGR and PE exhibit higher blood levels of hCG from early pregnancy until before delivery^9–11^, and placental dysfunction may reflect the increased hCG levels^12–15^. Therefore, we speculated that high hCG is a risk factor for the development of FGR and PE.

Mitochondria are essential organelles in eukaryotic cells. The main mitochondrial function is aerobic ATP synthesis via oxidative phosphorylation (OXPHOS). In addition, mitochondria produce and regulate metabolites and reactive oxygen species (ROS), regulate calcium ions and apoptosis, and have a crucial role in producing energy in STB^16,17^. The p32/complement component 1q binding protein (C1QBP) predominantly localizes in mitochondria^18^ is important for functional maturation in many cell types^19,20^. It has been reported that placental levels of p32/C1QBP (hereafter p32) tended to decrease in patients with FGR, and p32 is important for cytotrophoblast proliferation^21^. An association between PE and mitochondrial dysfunction has been reported. The expression of various OXPHOS complexes, such as complexes I–IV and cytochrome c oxidase (COX), and ATP were found to be decreased in placental samples from PE^22,23^. These reports suggested that mitochondrial translational dysfunction is involved in FGR and PE/FGR.

Angiopoietin-2 (Ang2), interferon gamma-inducible protein-10 (IP10), and soluble fms-like tyrosine kinase-1 (sFlt1) are antiangiogenic factors and elevated levels of these factors lead to endothelial dysfunction, which destabilizes the vasculature^24–26^. Tumor necrosis factor-alpha (TNFα), interleukin-8 (IL8), and interleukin-1 beta (IL1B) are triggers for tissue inflammation. It has been reported that TNFα, IL8, IL1B, and sFlt1 may reduce placental function^24,27–30^ and Ang2, IP10, sFlt1, and TNFα are elevated in the maternal blood of patients experiencing FGR and PE^31–34^.

It has been suggested that hypoxia plays an important role in the pathogenesis of PE and FGR. Hypoxia-inducible factors (HIFs) are important molecules that regulate the cellular response to hypoxia and play an important role in physiological and pathophysiological contexts in FGR and PE^35^. However, the relationship between hypoxia, HIF1ɑ stabilization, hCG, and the expression of various cytokines in the pathogenesis of FGR and PE/FGR are unclear.

In this study, we hypothesized that mitochondrial translation dysfunction and high hCG may be involved in the mechanism of onset of FGR and PE/FGR. To the best of our knowledge, this is the first report to show that mitochondrial translational disorder and high hCG levels are closely related to the pathological mechanisms of FGR and PE/FGR.

## Results

### Increased placental expression of hCGβ and GDF15 in patients with FGR and PE/FGR

**Figure 1:**
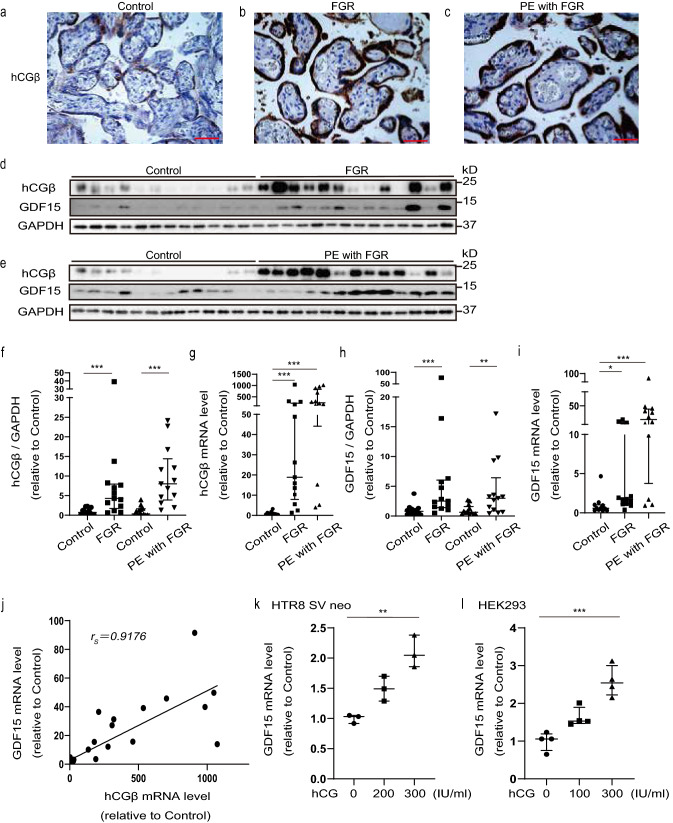
Increased expression of hCGβ and GDF15 in FGR and PE/FGR placental samples. (**a**–**c**) Placental sections from controls (**a**, 31th weeks of pregnancy), and patients with fetal growth restriction (FGR) (**b**, 32th weeks of pregnancy), and pre-eclampsia (PE) with FGR (**c**, 32th weeks of pregnancy) were analyzed by immunohistochemistry with anti-human chorionic gonadotropin-beta (hCGβ) (brown). Scale bars = 50 µm. (**d**,**e**) Western blot analysis of hCGβ and growth differentiation factor 15 (GDF15) protein in placentas from controls, FGR, and PE/FGR. The same control samples were used in the blots shown. GAPDH was used as an internal control. Controls, n = 13; FGR, n = 13; PE/FGR, n = 13. The full unedited gels are shown in the Supplementary information (Full unedited gel for Fig. 1). (**f**,**h**) Western blot quantification of placental hCGβ and GDF15. Values are presented as the median with an interquartile range. The Mann–Whitney test was performed on controls vs FGR and controls vs PE/FGR. **p < 0.01, ***p < 0.001. (**g**,**i**) Placental expression of hCGβ and *GDF15* mRNA in control, FGR, and PE/FGR samples. Values are presented as the median with an interquartile range. Controls, n = 11; FGR, n = 13; PE/FGR, n = 12. Statistical significance was assessed by the Kruskal–Wallis test with a Dunn’s multiple comparisons test. *p < 0.05, ***p < 0.001. (**j**) Correlation diagram of placental hCGβ and *GDF15* mRNA expression. Total, n = 36; controls, n = 11; FGR, n = 13; PE/FGR, n = 12. r_s_ = 0.9176, p < 0.001, and Spearman’s rank correlation test. (**k**,**l**) *GDF15* mRNA expression in HTR8 SV neo (**k**) and HEK293 (**l**) cells after hCG treatment for 48 h. Values are presented as the median with an interquartile range of three and four independent experiments, respectively. Statistical significance was assessed by the Kruskal–Wallis test. **p < 0.01, ***p < 0.001.

In previous reports, high hCG in maternal blood has been observed from early pregnancy in patients with FGR and PE^9–11^. Therefore, we first investigated whether hCG was also elevated in the postnatal placentas of these patients. Immunostaining showed elevated hCGβ in FGR and PE/FGR samples (Fig. 1a–c and Supplementary Fig. 1a–c). Western blotting showed that hCGβ protein was approximately 7.5- and 9.8-fold higher in FGR and PE/FGR samples, respectively, than in control samples (Fig. 1d–f). Expression of hCGβ mRNA was also elevated in FGR and PE/FGR samples (Fig. 1g), suggesting that the high hCG expression was transcriptionally regulated in placentas from patients showing FGR and PE/FGR.

To investigate whether mitochondrial dysfunction occurred in both diseases, we evaluated GDF15, which is a biomarker of mitochondrial dysfunction^36^. On western blotting, the expression of GDF15 protein was approximately 9.5- and 4.3-fold higher in placentas from patients with FGR and PE/FGR, respectively, than in placentas from normal pregnancy (Fig. 1d,e,h). *GDF15* mRNA expression was also markedly elevated (Fig. 1i), suggesting the presence of placental mitochondrial dysfunction.

Furthermore, there was a strong correlation between hCGβ and *GDF15* mRNA expression (Fig. 1j). We then investigated whether hCG induced *GDF15* mRNA expression in vitro. We confirmed that the luteinizing hormone/choriogonadotropin receptor (the hCG receptor) was expressed in HTR8 SV neo, HEK293, JEG3, and THP-1 cells by RT-PCR (Supplementary Fig. 1d). *GDF15* mRNA expression increased after hCG treatment in HTR8 SV neo and HEK293 cells (Fig. 1k,l), suggesting that high hCG expression interacts with mitochondrial dysfunction.

### High expression of antiangiogenic factors and inflammatory cytokines in placentas from patients with FGR and PE/FGR

**Figure 2:**
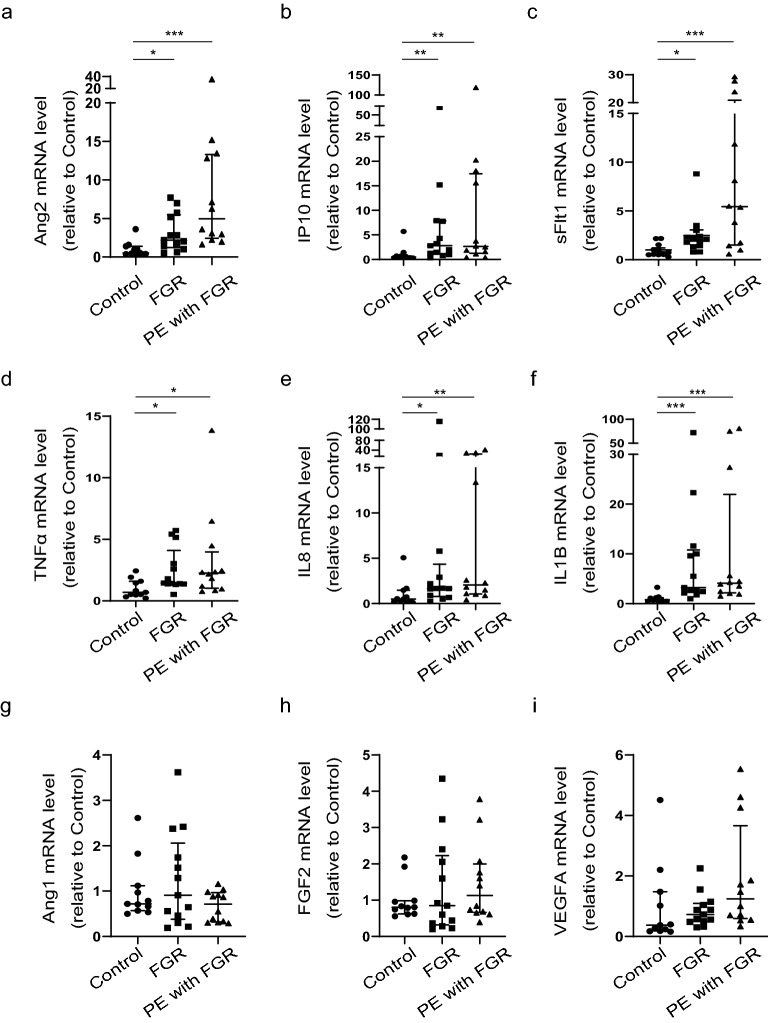
High expression of antiangiogenic factors and inflammatory cytokines in FGR and PE/FGR placentas. The figures show placental mRNA expression in control, fetal growth restriction (FGR), and pre-eclampsia (PE) with FGR samples. Controls, n = 11; FGR, n = 13; PE/FGR, n = 12. (**a**–**c**) Placental expression of antiangiogenic factors Ang2, IP10, and sFlt1. (**d**–**f**) Placental expression of inflammatory cytokines TNFα, IL8, and IL1B. (**g**–**i**) Placental expression of angiogenetic factors Ang1, fibroblast growth factor 2 (FGF2), and vascular endothelial growth factor A (VEGFA). (**a–i**) Values are presented as the median with an interquartile range. Statistical significance was assessed by the Kruskal–Wallis test with a Dunn’s multiple comparisons test. *p < 0.05, **p < 0.01, ***p < 0.001.

To examine the factors that influence placental function, we investigated antiangiogenic factors and inflammatory cytokine expression. We found that Ang2, IP10, sFlt1, TNFα, IL8, and IL1B mRNAs were overexpressed in both diseases (Fig. 2a–f). Expression of some angiogenic factors, such as angiopoietin-1 (Ang1), fibroblast growth factor 2 (FGF2), and vascular endothelial growth factor A (VEGFA) mRNAs were not increased (Fig. 2g–i), indicating that angiogenesis may be suppressed and tissue inflammation may be enhanced.

### hCG induced the expression of antiangiogenic factors and inflammatory cytokines via the p38 and JNK pathways

**Figure 3:**
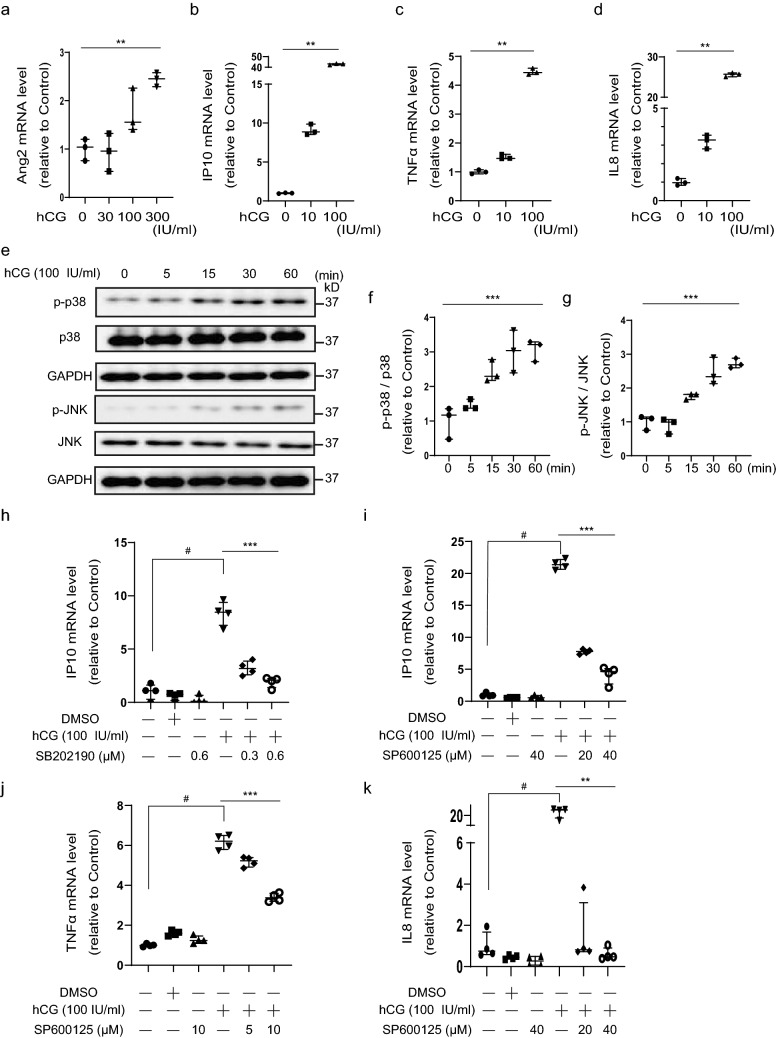
hCG induced the expression of antiangiogenic factors and inflammatory cytokines via the p38 and JNK pathway. (**a**–**d**) Ang2, IP10, TNFα, and IL8 mRNA expression in HEK293 (**a**) and THP-1 (**b**–**d**) cells after human chorionic gonadotropin (hCG) treatment for 48h (**a**) or 2 h (**b**–**d**). (**e**) Immunoblot analysis of p-p38, p38, p-JNK, and JNK expression in THP-1 cells after hCG treatment for 0, 5, 15, 30, and 60 min. The full unedited gels are shown in the Supplementary information (Full unedited gel for Fig. 3). (**f**,**g**) Western blot quantification of p-p38/p38 and p-JNK/JNK levels. (**h**–**k**) IP10, TNFα, and IL8 mRNA expression in THP-1 cells after incubation with hCG and/or p38 inhibitor (**h**; SB202190) and JNK inhibitor (**i**–**k**; SP600125) for 2 h. (**a**–**d**, **f**–**k**) Values are presented as the median with an interquartile range of three or four independent experiments. Statistical significance was assessed by the Kruskal–Wallis test (*) and the Mann–Whitney test (#). **p < 0.01, ***p < 0.001, #p < 0.05.

To explore how hCG increased the expression of antiangiogenic factors and inflammatory cytokines, we investigated gene expression after hCG treatment of the JEG3, HEK293, and THP-1 cell lines. We used HEK293 cells for hCG treatment because the expression of *GDF15* mRNA expression was found to be increased after hCG treatment in HEK293 cells (Fig. 11). Furthermore, since PE has been reported to be characterized by proteinuria^2^, we used HEK293 cells to investigate the effects of hCG on the kidney. The monocytic THP-1 cell line was examined because cytokines are released by monocytes surrounding the placenta. hCG stimulation significantly upregulated Ang2 mRNA expression in HEK293 cells in a dose-dependent manner (Fig. 3a). We also observed IP10, TNFα, and IL8 mRNA expression in THP-1 cells (Fig. 3b–d), and TNFα and IL8 mRNA in JEG3 cells (Supplementary Fig. 1e,f). These results suggest that high levels of hCG may have adverse effects on systemic organs such as the kidneys, monocytes, and placenta.

hCG induces the activation of mitogen-activated protein kinases (MAPKs) and c-Jun N-terminal kinase (JNK) signaling cascades and contributes to steroid synthesis and gene expression^37–39^. We found that hCG induced p38 MAPK and JNK phosphorylation in a time-dependent manner in THP-1 cells (Fig. 3e–g), and p38 phosphorylation was elevated in placentas from both disease states (Supplementary Fig. 1g,h). Next, we found that p38 and JNK inhibitors significantly inhibited hCG-induced IP10 mRNA expression in THP-1 cells (Fig. 3h,i). Also, hCG-induced TNFα and IL8 mRNA expression were decreased by a JNK inhibitor (Fig. 3j,k). These results suggest that p38 and JNK phosphorylation are involved in the hCG-induced cytokine mRNA expression.

### Reduced mitochondrial translation in placentas from patients with FGR and PE/FGR

**Figure 4:**
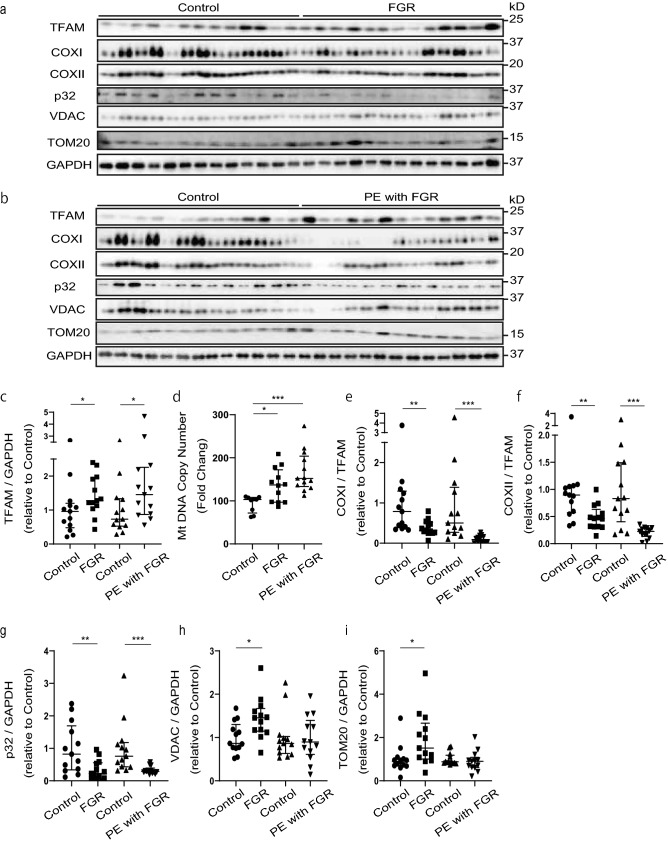
Reduced mitochondrial translation in FGR and PE/FGR placentas. (**a**,**b**) Western blot analysis of placental expression of transcription factor A/mitochondrial (TFAM), COXI, COXII, p32, VDAC and TOM20 in control, fetal growth restriction (FGR), and pre-eclampsia (PE) with FGR samples. The same control samples were used in the blots shown. Controls, n = 13; FGR, n = 13; PE/FGR, n = 13. The full unedited gels are shown in the Supplementary information (Full unedited gel for Fig. 4). (**c**,**g**) Western blot quantification of TFAM and p32. GAPDH was used as an internal control. (**d**) The mtDNA copy number in control, FGR, and PE/FGR samples. Controls, n = 9; FGR, n = 12; PE/FGR, n = 13. The mtDNA count was measured by RT-qPCR normalizing the quantity of the *HPRT* gene. (**e**,**f**) Western blot quantification of COXI/TFAM and COXII/TFAM. (**h**,**i**) Western blot quantification of placental VDAC and TOM20. GAPDH was used as an internal control. (**c**,**e–i**) Values are presented as the median with an interquartile range. The Mann–Whitney test was performed on controls vs FGR and controls vs PE/FGR. *p < 0.05, **p < 0.01, ***p < 0.001. (**d**) Values are presented as the median with an interquartile range. Statistical significance was assessed by the Kruskal–Wallis test with a Dunn’s multiple comparisons test. *p < 0.05, ***p < 0.001.

We examined why hCG and inflammatory cytokines were induced in placentas associated with FGR and PE/FGR. We hypothesized that mitochondrial homeostasis—such as mitochondrial replication, transcription, and translation—might be altered, affecting hCG and inflammatory cytokines. First, we evaluated whether mitochondrial replication, translation, and transcription were changed. Transcription factor A/mitochondrial (TFAM), which binds to mitochondrial DNA (mtDNA) and whose expression is correlated with mtDNA levels^40^, was increased in placental samples from patients with FGR and PE/FGR, compared with controls (Fig. 4a–c). Inevitably, we found that the mtDNA copy number was increased in placental samples from patients with FGR and PE/FGR compared with controls (Fig. 4d). We observed no change in the expression levels of mRNA encoded by the mtDNA (Supplementary Fig. 2a–e), which suggests that mitochondrial replication and RNA transcription were not decreased in both diseases.

Next, we examined mitochondrial DNA-encoded COXI and COXII protein per TFAM expression in the placenta and found that they were significantly decreased in both diseases, suggesting that mitochondrial translation efficiency is reduced in placental samples associated with FGR and PE/FGR. (Fig. 4a,b,e,f, Supplementary Fig. 2f–k, and Supplementary Fig. 3a–f). Then, we found that the expression of p32 which is involved in mitochondrial translation was also reduced. (Fig. 4a,b,g, Supplementary Fig. 2l, m, and Supplementary Fig. 3g,h).

We considered the possibility that the decreased expression of COXI and COXII resulted from a decrease in mitochondria. Therefore, we measured the mitochondrial outer membrane proteins, voltage-dependent anion channel (VDAC), and the translocase of outer mitochondrial membrane 20 (TOM20). Elevated expression levels of VDAC and TOM20 proteins were observed in placentas from patients with FGR, but there were no changes in expression in samples associated with PE/FGR, indicating that mitochondrial translational dysfunction was not caused by reduced mitochondrial number or mass (Fig. 4a,b,h,i). These results suggest that the high expression of hCG and mitochondrial translational dysfunction may play an important role in the pathogenesis of FGR and PE/FGR (Figs. 1, 2, 3, 4, and Supplementary Fig. 1–3).

### Mitochondrial translation deficiency induced expression of hCGβ and inflammatory cytokine factors

**Figure 5:**
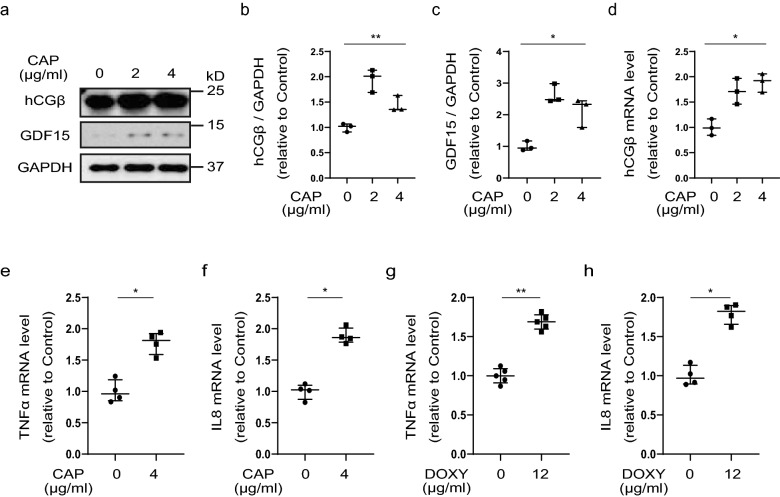
Mitochondrial translation deficiency induced expression of hCGβ and inflammatory cytokine factors. (**a**) Immunoblot analysis of human chorionic gonadotropin-beta (hCGβ) and growth differentiation factor 15 (GDF15) in JEG3 cells after chloramphenicol (CAP) treatment for 72 h. GAPDH was used as an internal control. The full unedited gels are shown in the Supplementary information (Full unedited gel for Fig. 5). (**b**,**c**) Western blot quantification of hCGβ and GDF15. (**d**) hCGβ mRNA expression in JEG3 cells after CAP treatment for 72 h. (**e**–**h**) TNFα and IL8 mRNA expression in JEG3 cells after CAP (**e**,**f**) and doxycycline (DOXY; **g**,**h**) treatment for 72 h. (**b**–**d**) Values are presented as the median with an interquartile range of three independent experiments. Statistical significance was assessed by the Kruskal–Wallis test. *p < 0.05, **p < 0.01. (**e**–**h**) Values are presented as the median with an interquartile range of four or five independent experiments. Statistical significance was assessed by Mann–Whitney test. *p < 0.05, **p < 0.01.

Next, we investigated whether the mitochondrial translation defect induced the expression of hCGβ and inflammatory cytokine genes. We used chloramphenicol (CAP) and doxycycline (DOXY) to inhibit mitochondrial translation in vitro. We observed that mitochondrial translation inhibitors (CAP and DOXY) induced hCGβ and GDF15 protein expression in JEG3 cells (Fig. 5a–c, and Supplementary Fig. 4a–c) and activated hCGβ mRNA expression in a dose-dependent manner (Fig. 5d, and Supplementary Fig. 4f). The mitochondrial translation inhibitors also induced TNFα and IL8 mRNA expression in JEG3 cells (Fig. 5e–h). We showed that the mitochondrial translation inhibitors decreased COXI and COXII protein expression in JEG3 cells and another trophoblast cell line, HTR8/SV neo (Supplementary Fig. 4a,d,e,g–n). These results suggest that mitochondrial translation deficiency induced hCGβ gene expression, which led to the expression of inflammatory cytokines.

### Impairment of mitochondrial translation contributed to elevation of hCGβ via HIF1α

**Figure 6:**
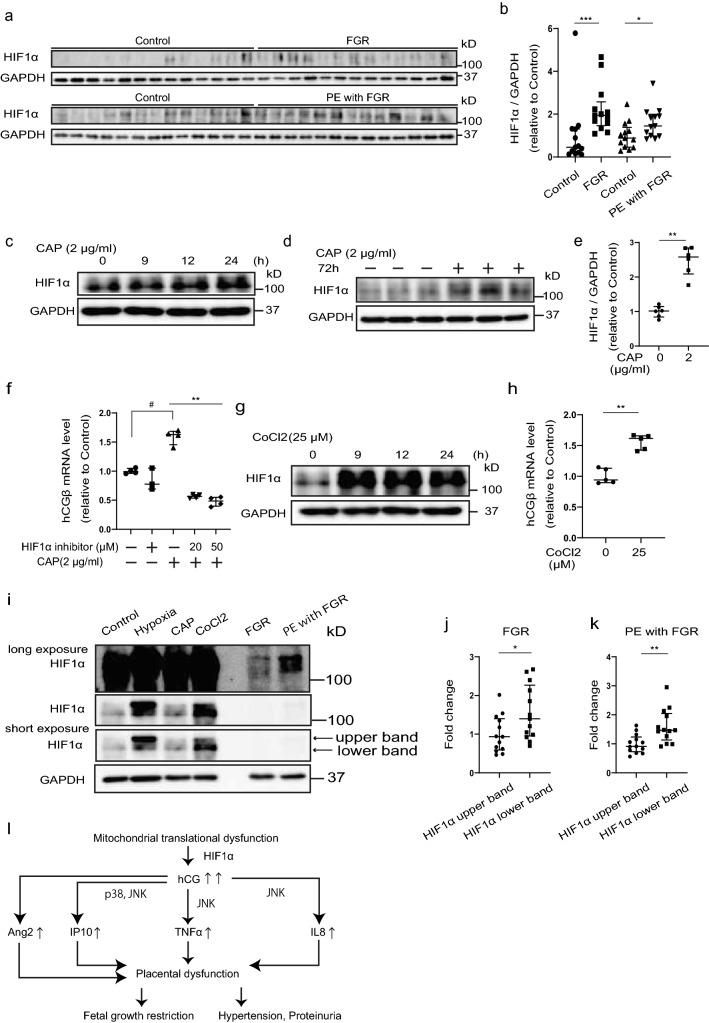
Impairment of mitochondrial translation contributed to elevation of hCGβ via HIF1α. (**a**) Western blot analysis of placental expression of HIF1α protein in control, fetal growth restriction (FGR), and pre-eclampsia (PE) with FGR samples. GAPDH was used as an internal control. The same control samples were used in the blots shown. Controls, n = 13; FGR, n = 13; PE/FGR, n = 13. (**b**) Western blot quantification of HIF1α. Values are presented as the median with an interquartile range. The Mann–Whitney test was performed on controls vs FGR and controls vs PE/FGR. *p < 0.05, ***p < 0.001. (**c**) Immunoblot analysis of HIF1α in JEG3 cells after chloramphenicol (CAP) treatment for 0, 9, 12, and 24 h. (**d**) Immunoblot analysis of HIF1α in JEG3 cells after CAP treatment for 72 h. GAPDH was used as an internal control. (**e**) Western blot quantification of HIF1α. Values are presented as the median with an interquartile range. Statistical significance was assessed by Mann–Whitney test. **p < 0.01. (**f**) hCGβ mRNA expression in JEG3 cells after treatment with CAP and/or HIF1α inhibitor for 72 h. Values are presented as the median with an interquartile range of three or four independent experiments. Statistical significance was assessed by the Kruskal–Wallis test (*) and the Mann–Whitney test (#). **p < 0.01, #p < 0.05. (**g**) Immunoblot analysis of HIF1α in JEG3 cells after CoCl_2_ treatment for 0, 9, 12, and 24 h. GAPDH was used as an internal control. (**h**) hCGβ mRNA expression in JEG3 cells after CoCl_2_ treatment for 72 h. Values are presented as the median with an interquartile range of five independent experiments. Statistical significance was assessed by Mann–Whitney test. **p < 0.01. (**i**) Immunoblot analysis of HIF1α and GAPDH in FGR, PE with FGR, and JEG3 cells after the treatment of hypoxia, CAP, and CoCl_2_ for 24 h. (**j**,**k**) Western blot quantification of upper and lower bands of HIF1α (**a**) in FGR and PE with FGR. FGR n = 13; PE/FGR, n = 13. Values are presented as the median with an interquartile range. The Mann–Whitney test was performed on the HIF1α upper band vs HIF1α lower band. *p < 0.05, **p < 0.01. (**l**) This scheme shows our proposed novel mechanism for the development of FGR and PE/FGR. (**a**,**c**,**d**,**g**,**i**) The full unedited gels are shown in the Supplementary information (Full unedited gel for Fig. 6).

We investigated the mechanism involved in the induction of hCGβ gene expression by mitochondrial translation deficiency. We focused on the transcription factor hypoxia-inducible factor 1-alpha (HIF1α), which was upregulated in placentas associated with FGR and PE/FGR (Fig. 6a,b). We observed that CAP induced HIF1α expression in JEG3 cells in a time‐dependent manner (Fig. 6c). After 72 h treatment with CAP, HIF1α levels stabilized (Fig. 6d,e). Moreover, CAP treatment induced hCGβ expression (Fig. 6f), and CoCl_2_ treatment, which stabilized HIF1α expression (Fig. 6g), also induced hCGβ gene expression (Fig. 6h). Conversely, the HIF1α inhibitor suppressed the CAP-induced expression of hCGβ (Fig. 6f), which suggests that inhibition of mitochondrial translation induced HIF1α expression, which led to activation of hCGβ expression.

Both FGR and PE have been reported to be derived from the placenta by gestational hypoxia^41^. We investigated whether hCG was induced under hypoxic conditions in JEG3. However, we found that hypoxia reduced COXI expression, other mitochondrial proteins, and hCGβ expression (Supplementary Fig. 5a).

The band position of HIF1α was different between hypoxia and CAP, CoCl_2_ treatment in JEG3, suggesting that the post-translational modification of HIF1α is different (Fig. 6i). The expression of the lower band of HIF1α was increased in FGR and PE/FGR compared to the upper band, suggesting that the expression of HIF1α in both diseases is similar to that in CAP and CoCl_2_ treatment (Fig. 6i–k). We further examined whether hCG treatment affects p32 expression and tracks mitochondrial translation as a feedback mechanism. The results showed that hCG treatment did not change the protein expression levels of COXI, COXII, p32 or GDF15 in JEG3 (Supplementary Fig. 5b–f), suggesting that mitochondrial translational dysfunction, but not hypoxia or high hCG, may be upstream of the pathogenesis of FGR and PE/FGR.

## Discussion

Our working hypothesis is that in placentas associated with FGR and PE/FGR, mitochondrial dysfunction sustains high hCG expression via stabilization of HIF1ɑ, which leads to an increased expression of antiangiogenic factors and inflammatory cytokines, which subsequently may alter spiral artery remodeling to generate a prolonged ischemic state (Fig. 6l).

We propose that the persistence of high hCG levels due to mitochondrial dysfunction from early pregnancy provides a mechanism for the development of FGR and PE/FGR. In complete hydatidiform moles with coexistent fetus (CHMCF) with hCG-producing tumors, hCG levels are high from the beginning of pregnancy and patients develop hypertensive disorders of pregnancy (HDP) at a high rate^42^. These cases suggest that high hCG in early pregnancy is involved in PE.

In addition, multiple placentas produce relatively more hCG than singleton pregnancies, which suggests that multiple pregnancies are more likely to develop HDP^43^. It has been reported that the hCG value at 13th–20th weeks of pregnancy is associated with the severity of HDP^44^. These observations also suggest that high hCG levels are important as a major etiological factor in PE.

Hypoxia induced the stabilization of HIF1ɑ, mitochondrial dysfunction, but hCGβ expression was not increased, the result consistent with previous reports^45–47^. hCGβ expression was induced in the treatment of CAP and CoCl_2_ but not in the hypoxia condition. This difference may be related to post-translational modifications of HIF1α. HIF1α is subjected to post-translational modifications such as hydroxylation, ubiquitination, acetylation, and sumoylation which are related to the regulation of its stability. The small ubiquitin-related modifier-1 (SUMO-1) is around 12 kDa protein that is covalently linked to a lysine residue of consensus motif. Sumoylation is induced by hypoxia, is involved in protein stabilization and transcriptional regulation, and enhances the transcriptional activity of HIF1α^48,49^. In addition, carbohydrate response element binding protein (ChREBP) also binds to the promoter region of HIF1α and regulates downstream gene expression^50^. These reports suggest that post-translational modification of HIF1α alters the regulation of downstream transcription factors. The difference in post-translational modification may be related to the induction of hCG.

Why was mitochondrial translation impaired in placentas associated with FGR and PE/FGR? We speculate that mitochondrial dysfunction of STB may disrupt their antioxidant function in early pregnancy. STB have almost no Cu/Zn superoxide dismutase and low antioxidant capacity until 8th weeks of pregnancy. However, as the expression of Cu/Zn superoxide dismutase gradually increases in STB from 10 to 14th weeks, when placental blood flow has increased, the STB acquire antioxidant functions for ROS^51–53^. In the current study, it is possible that the mitochondrial function of the STB may be reduced by the production of ROS during a state when the antioxidant function cannot be appropriately acquired for some factors.

Since FGR is a heterogeneous population, our proposed mechanism will not apply to all FGR cases. However, cases where HDP develops after the onset of FGR have been clinically well reported^54^. It is possible that our unexplained FGR samples may be the FGR cases that delivered before the onset of HDP and therefore may have been a population with a similar background to PE/FGR. We also believe that there is a subset of the FGR population that has a similar etiology to PE/FGR.

Our study examined many placental samples, and this is the first paper to propose that mitochondrial dysfunction and high hCG may reduce placental function. Previous reports speculated that mitochondrial dysfunction occurs in early pregnancy, but the current study did not examine this period and provides no direct evidence that mitochondrial dysfunction and high hCG cause both diseases. This will be the subject of future studies, such as animal studies.

Our studies have indicated that continuous high hCG levels and a mitochondrial translational disorder may directly reduce placental function. Our data provide a new mechanism for the development of both FGR and PE/FGR and may provide novel avenues and potential targets for the treatment of these conditions.

## Methods

### Patients

Patients were Japanese women who had a prenatal check and a singleton birth at Kyushu University Hospital between 2014–2019. Clinical characteristics of the patients are shown in Tables 1 and 2. FGR is defined as an estimated fetal weight below − 1.5 SD or less on ultrasonography and small for gestational age after birth. We used preterm placentas as a control, because hCG and other cytokines have been reported to change during pregnancy^55,56^. In our experiment, gestational weeks for controls, FGR, and PE/FGR were matched to minimize the effect of these changes. The reasons for preterm birth of controls were placenta previa, pregnancy with cervical cancer, and threatened premature delivery. Samples with fetal morphological abnormalities, chromosomal abnormalities, and pathological placental infection were excluded. There were no significant differences in maternal age, gestational age at delivery, delivery mode, and sex of the baby between each group. Placenta samples were collected as soon as possible after delivery, and placental tissue just below the umbilical cord attachment was dissected at 1 cm × 1 cm × 1 cm. After that, the chorion and decidua were removed and stored at − 80 °C. These samples were stored at − 80 °C for protein and mRNA assays and fixed in formamide for immunohistochemistry. This study was approved by the Human Genome/Gene Analysis Research Ethical Committee of Kyushu University (#731-02) and we obtained informed consent from all patients and carried out in accordance with the Declaration of Helsinki. All methods were performed in accordance with the relevant guidelines and regulations.

**Table 1:** Clinical characteristics of the pregnancies in the western blot.

	Control	FGR	PE with FGR
N	13	13	13
Maternal age (years)	32 (19–38)	31 (22–43)	32 (26–41)
Gestational age at delivery (weeks)	31.9 (23.7–35.6)	34.1 (26.7–36.6)	30.6 (25.9–36.4)
Birth weight (g)	2052 (706–2520)	1377 (476–2166)*	912 (472–1456)***
SBP (mmHg)	127 (108–134)	132 (100–138)	166 (140–180)***
DBP (mmHg)	74 (52–85)	84 (54–88)	104 (80–117)***
24 h-protein excretion (g/24 h)			5.4 (1.1–12.5)
**Sex of the baby**			
Male/female	9/4	8/5	5/8
**Delivery mode**			
VD	3	0	0
C/S	10	13	13

Data presented as median (range). The Mann–Whitney test was performed on controls vs FGR and controls vs PE/FGR in maternal age, gestational age at delivery, birth weight, SBP, and DBP. *p < 0.05, ***p < 0.001. The Fisher’s exact test was performed on controls vs FGR and controls vs PE/FGR for sex of the baby and delivery mode.

*FGR* fetal growth restriction, *PE* pre-eclampsia, *SBP* systolic blood pressure, *DBP* diastolic blood pressure, *VD* vaginal delivery, *C/S* Caesarean section.

**Table 2:** Clinical characteristics of the pregnancies in the RT-qPCR.

	Control	FGR	PE with FGR
N	11	13	12
Maternal age (years)	35 (29–37)	30 (22–38)	32 (25–43)
Gestational age at delivery (weeks)	31.9 (26.6–35.3)	33.6 (26.7–36.3)	30.8 (24.4–32.4)
Birth weight (g)	2054 (874–2538)	1242 (424–1814)**	877 (426–1414)***
SBP (mmHg)	127 (108–134)	131 (108–138)	170 (160–184)***
DBP (mmHg)	74 (52–88)	83 (70–88)	100 (80–123)***
24 h-protein excretion (g/24 h)			5.1 (0.3–11)
**Sex of the baby**			
Male/female	8/3	7/6	5/7
**Delivery mode**			
VD	3	0	0
C/S	8	13	12

Data presented as median (range). The Mann–Whitney test was performed on controls vs FGR and controls vs PE/FGR for maternal age, gestational age at delivery, birth weight, SBP, and DBP. **p < 0.01, ***p < 0.001. The Fisher’s exact test was performed on controls vs FGR and controls vs PE/FGR for sex of the baby and delivery mode.

*FGR* fetal growth restriction, *PE* pre-eclampsia, *SBP* systolic blood pressure, *DBP* diastolic blood pressure, *VD* vaginal delivery, *C/S* Caesarean section.

### Cell culture

JEG3 (HTB-36) and HEK293 cell lines were cultured in Dulbecco’s modified Eagle medium (DMEM medium, Sigma-Aldrich, St. Louis, USA) containing 10% fetal bovine serum (FBS; Sigma-Aldrich, USA), and 5% penicillin–streptomycin (Nacalai Tesque, Japan). HTR8 SV/neo (American Type Culture Collection, USA) and THP-1 cells were grown in RPMI 1640 medium (Sigma-Aldrich, USA) supplemented with the same supplements as above. The cell lines were cultured in a humidified incubator with 95% air and 5% CO_2_ at 37 °C.

### Quantitative real-time PCR

Total RNA of cell lines and placentas were extracted with the RNeasy Mini Kit (QIAGEN, Germany) and ReliaPrep™ RNA Tissue Miniprep systems (Promega, USA), respectively. RNA samples were reverse transcribed using the PrimeScript™ RT Reagent Kit (TAKARA, Japan) according to the manufacturer’s instructions. mRNA expression was detected by qPCR with a thermal cycler (Step One plus; Applied Biosystems). Ribosomal 18S rRNA was evaluated as an internal control. Primer sequences are shown in Table 3.

**Table 3:** List of primers.

Primer pair	Forward	bp	Reverse	bp
hCGβ	gcttcagtccagcacctttc	20	cacggtgaagtgacctcaga	20
GDF15	ctccagattccgagagttgc	20	agagatacgcaggtgcaggt	20
Ang1	gaagggaaccgagcctattc	20	gggcacatttgcacatacag	20
Ang2	gcaagtgctggagaacatca	20	gttaacttccgcgtttgctc	20
IP10	ctgtacgctgtacctgcatca	20	ttcttgatggccttcgattc	20
sFlt1	aggggaagaaatcctccaga	20	tcctccgagcctgaaagtta	20
TNFα	cagagggcctgtacctcatc	20	ggaagacccctcccagatag	20
IL8	gtgcagttttgccaaggagt	20	ctctgcacccagttttcctt	20
IL1B	gggcctcaaggaaaagaat	20	ttctgcttgagaggtgctga	20
FGF2	agagcgaccctcacatcaa	20	actgcccagttcgtttcagt	20
VEGFA	cccactgaggagtccaacat	20	tttcttgcgctttcgttttt	20
COX1	ggcctgactggcattgtatt	20	tggcgtaggtttggtctagg	20
COX2	ttcatgatcacgccctcata	20	taaaggatgcgtagggatgg	20
COX3	cccgctaaatcccctagaag	20	ggaagcctgtggctacaaaa	20
ND1	atggccaacctcctactcct	20	gcggtgatgtagagggtgat	20
CYTB	tatccgccatcccatacatt	20	ggtgattcctagggggttgt	20
LHCGR	tcaattcttgtgccaatcca	20	ccatttttgcagttggaggt	20
Human mtDNA	cgatgttggatcaggacatc	20	aaggcgctttgtgaagtagg	20
HPRT	cctggggattccaaatacct	20	gggcagaaaaggtcatcaaa	20

### MtDNA copy number assay

Total DNA from placental tissue was extracted with the NucleoSpin Tissue kit (MACHEREY–NAGEL, Germany). The mtDNA content was measured by real-time qPCR with a thermal cycler (Step One plus; Applied Biosystems). HPRT was evaluated as an internal control. Primer sequences are shown in Table 3.

### Antibodies

Antibodies to p32 (1:5000) and TFAM (1:5000) were raised in our laboratory. Antibodies to COXI (1:5000, ab14705), COXII (1:5000, ab110258), hCGβ (1:1000, ab53087), VDAC (1:5000, ab14734), and HIF1α (1:5000, ab179483) were purchased from Abcam. Antibodies to GDF15 (1:5000, 3209s), p-p38 (1:5000, 4631s), p38 (1:5000, 9212P), JNK (1:5000, 9258P), p-JNK (1:5000, 4668P), and GAPDH (1:5000, 2118S), and anti-rabbit IgG HRP-linked antibody (1:5000, 7074S) and anti-mouse IgG HRP-linked antibody (1:5000, 7076S) were purchased from Cell Signaling Technology. Anti-TOM20 (1:5000, sc17764) was purchased from Santa Cruz.

### Immunoblotting analysis

Placental samples were homogenized in lysis buffer (20 mM Tris–HCl, pH 7.5, 2 mM EDTA, 150 mM NaCl and 1% NP40) containing protease inhibitor (FUJIFILM WAKO, 161-26021, Japan) and phosphatase inhibitors (Sigma-Aldrich, 4906837001, USA) and then centrifuged at 15,000×*g* for 15 min. The supernatants were collected as samples. Cell lines were also lysed with the same lysis buffer as above and after sonication were centrifuged at 15,000×*g* for 5 min. The supernatants were collected as samples. Equal amounts of protein (5 µg) were separated by SDS-PAGE and transferred to Immobilon-P Transfer Membranes (EMD Millipore Corporation, Germany).

Membranes were blocked using Blocking One (Nacalai Tesque, Japan) for 1 h at room temperature and then probed overnight with primary antibody at 4 °C. Membranes were incubated with secondary antibody in a low-temperature room for 2 h. Proteins were detected by enhanced chemiluminescence (GE Healthcare, Chalfont St. Giles, UK). Chemiluminescence was recorded and quantified with a chilled-charge-coupled device camera (LAS1000 plus).

### Hypoxic treatment

Hypoxic conditions were maintained in a personal CO_2_ multi gas incubator (ASTEC, Japan) with 1% O_2_, 5% CO_2_, and 94% NO_2_ at 37 °C. The O_2_ was monitored with an automatic gas mixer Gas Cylinder Auto Changer (Model 8420, WAKEN, Japan). Cultures in normoxic conditions were maintained in a humidified incubator with 95% air and 5% CO_2_ at 37 °C. JEG3 cell lines were separately cultured under the above hypoxic and normoxic conditions for 2, 3, and 4 days in DMEM medium before collection, and then we performed immunoblotting analysis as described above.

### Immunohistochemistry

Placental samples (3 µm thick) from 10% formalin-fixed, paraffin-embedded material were deparaffinized in xylene and dehydrated through ethanol solutions. Endogenous peroxidase activity was then blocked by methanol containing 0.3% hydrogen peroxidase for 30 min. The sections were incubated with anti-hCG (1:500, Dako, A0231) overnight at 4 °C and then incubated with Envision + Dual Link, Single Reagents, HRP Rabbit/Mouse (undiluted solution, Dako, K4063) for 30 min at room temperature. The reaction products were visualized by 3,3-diaminobenzidine tetrahydrochloride, and the sections were counterstained with hematoxylin.

### Drugs and materials

This study used hCG (Sigma-Aldrich, CG10-1VL, USA), CoCl_2_ (FUJIFILM WAKO, 036-03682, Japan), chloramphenicol (FUJIFILM WAKO, 034-10572, Japan), doxycycline (Sigma-Aldrich, D9891-5G, USA), p38 inhibitor (Funakoshi, SB202190, Japan), JNK inhibitor (Funakoshi, SP600125, Japan), and HIF1α inhibitor (Sigma-Aldrich, 400083, Canada). The concentration of each drug is shown in each figure. The normal hCG range in maternal blood during pregnancy has been reported to be approximately 10–190 IU/ml^55^. Therefore, hCG concentrations of 200 and 300 IU/ml were higher than the normal concentrations.

### Statistical analysis

Statistical analyses are described in each figure legend. Error bars are presented as the median with an interquartile range of the indicated number of experiments and placentas. Statistical analysis was performed using GraphPad Prism 8 (GraphPad Prism Software Inc). *p < 0.05, **p < 0.01, and ***p < 0.001 were considered statistically significant.

## Supplementary Information

Supplementary Information.

## Data Availability

All data generated or analysed during this study are included in this published article (and its Supplementary Information files).
