# Supplementary material for: Mitochondrial dysfunction-induced high hCG associated with development of fetal growth restriction and pre-eclampsia with fetal growth restriction
Source: Sci Rep. 2022 Mar 8;12:4056. doi: 10.1038/s41598-022-07893-y (PMC8904547; doi:10.1038/s41598-022-07893-y)

Title:

Mitochondrial dysfunction-induced high hCG associated with development of fetal growth restriction and preeclampsia with fetal growth restriction.

Ryo Kiyokoba[1,2], Takeshi Uchiumi[1,3*], Mikako Yagi[1,3], Takahiro Toshima[1], Shigehiro Tsukahara[1], Yasuyuki Fujita[2], Kiyoko Kato[2] and Dongchon Kang[1]

[1]Department of Clinical Chemistry and Laboratory Medicine, [2]Department of Obstetrics and Gynecology, [3]Department of Health Sciences, Graduate School of Medical Sciences, Kyushu University, Maidashi 3-1-1, Higashi-ku, Fukuoka 812-8582, Japan

*Correspondence: Dr. Takeshi Uchiumi: Department of Clinical Chemistry and Laboratory Medicine, Graduate School of Medical Sciences, Kyushu University, Maidashi 3-1-1, Higashi-ku, Fukuoka 812-8582, Japan :uchiumi@cclm.med.kyushu-u.ac.jp
TEL: +81-92-642-5750, FAX: +81-92-642-5772

Contents:

1. 4 Supplementary Figures (Supplementary Fig. 1-Fig. 4)
2. 7 Full unedited gels for Figures (Full unedited gel for Fig. 1, 3-6,Supplementary Fig.1,4)

Supplementary Fig. 1

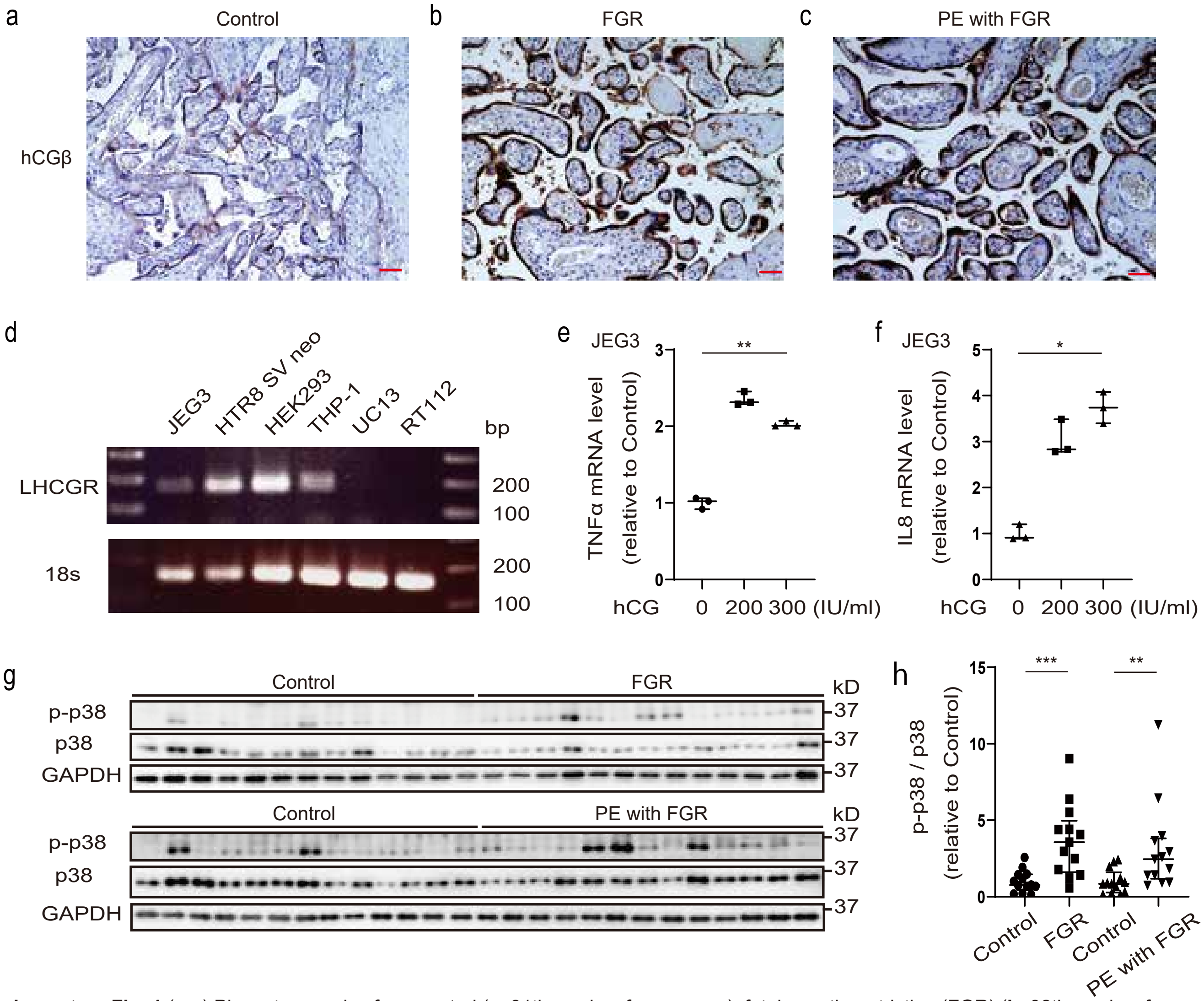

Supplementary Fig. 1 (**a-c**) Placenta samples from control (**a**; 31th weeks of pregnancy), fetal growth restriction (FGR) (**b**; 32th weeks of pregnancy), and preeclampsia (PE) with FGR (**c**; 32th weeks of pregnancy) samples were analyzed by immunohistochemistry using anti-human chorionic gonadotropin-beta (hCGβ) (brown). Scale bars=50 μm. (**d**) Expression of luteinizing hormone/choriogonadotropin receptor (LHCGR) mRNA, in JEG3, HTR8 SV neo, HEK293, THP-1, UC13, and RT112 cell lines analyzed by RT-PCR. For each lane, 0.8 μM of primer was used for RT. UC13 and RT112 cells are human bladder carcinoma cell lines. (**e, f**) TNFα and IL8 mRNA expression in JEG3 cells after hCG treatment for 72 h. Values are presented as the median with an interquartile range of three independent experiments. Statistical significance was assessed by the Kruskal–Wallis test.*$p<0.05$, **$p<0.01$. (**g**) Western blot analysis of placental expression of p-p38 and p38 protein in control, FGR, and PE with FGR samples. The same control samples were used in the blots shown. Control, n=13; FGR, n=13; PE with FGR, n=13. (**h**) Western blot quantification of p-p38/p38. Values are presented as the median with an interquartile range. The Mann–Whitney test was performed on control vs FGR and control vs PE with FGR. **$p<0.01$, ***$p<0.001$.
(**d**, **g**) The full unedited gels were shown in Supplementary information (Full unedited gel for Supplementary Fig.1).

Supplementary Fig. 2

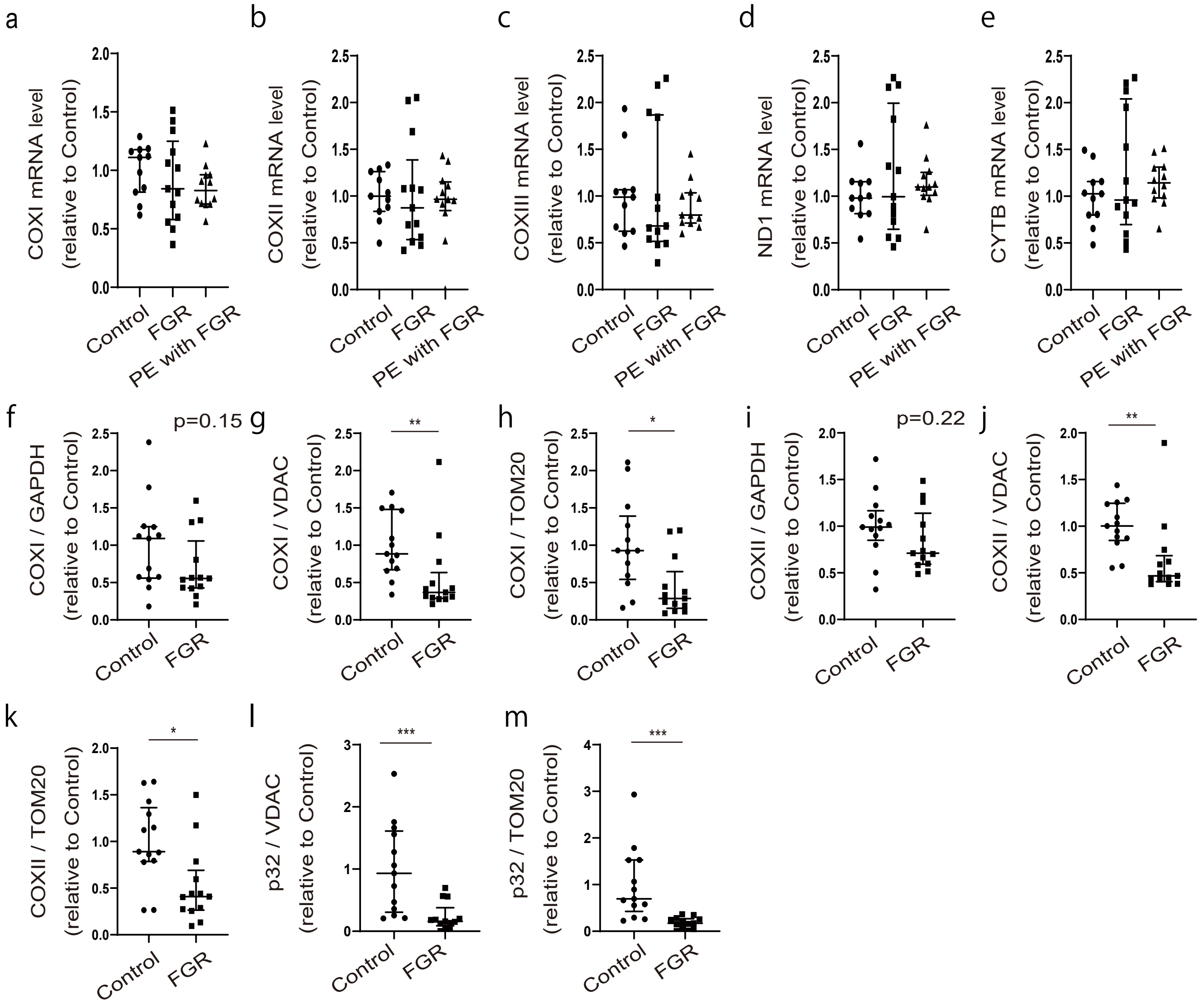

Supplementary Fig. 2 (**a-e**) Placental expression of COXI (**a**), COXII (**b**), COXIII (**c**), ND1 (**d**), and CTYB (**e**) mRNA levels, which are encoded by mtDNA. The mRNA levels were equivalent in control, fetal growth restriction (FGR), and preeclampsia (PE) with FGR samples. Values are presented as the median with an interquartile range. Control, n=11; FGR, n=13; PE with FGR, n=12. Statistical significance was assessed by the Kruskal–Wallis test with a Dunn' s multiple comparisons test. (**f**) Western blot quantification of placental COXI. GAPDH was used as an internal control. (**g**,**h**) Western blot quantification of COXI/VDAC and COXI/TOM20. COXI/VDAC and COXI/TOM20 indicate the amount of COXI per mitochondria.(**i**) Western blot quantification of placental COXII. GAPDH was used as an internal control. (**j**, **k**) Western blot quantification of COXII/VDAC and COXII/TOM20. COXII/VDAC and COXII/TOM20 indicate the amount of COXII per mitochondria. (**l**, **m**) Western blot quantification of p32/VDAC and p32/TOM20. p32/VDAC and p32/TOM20 indicate the amount of p32 per mitochondria.(**f-m**) Values are presented as the median with an interquartile range. Control, n=13; FGR, n=13. The Mann–Whitney test was performed on control vs FGR. *p<0.05, **p<0.01, ***p<0.001

Supplementary Fig. 3

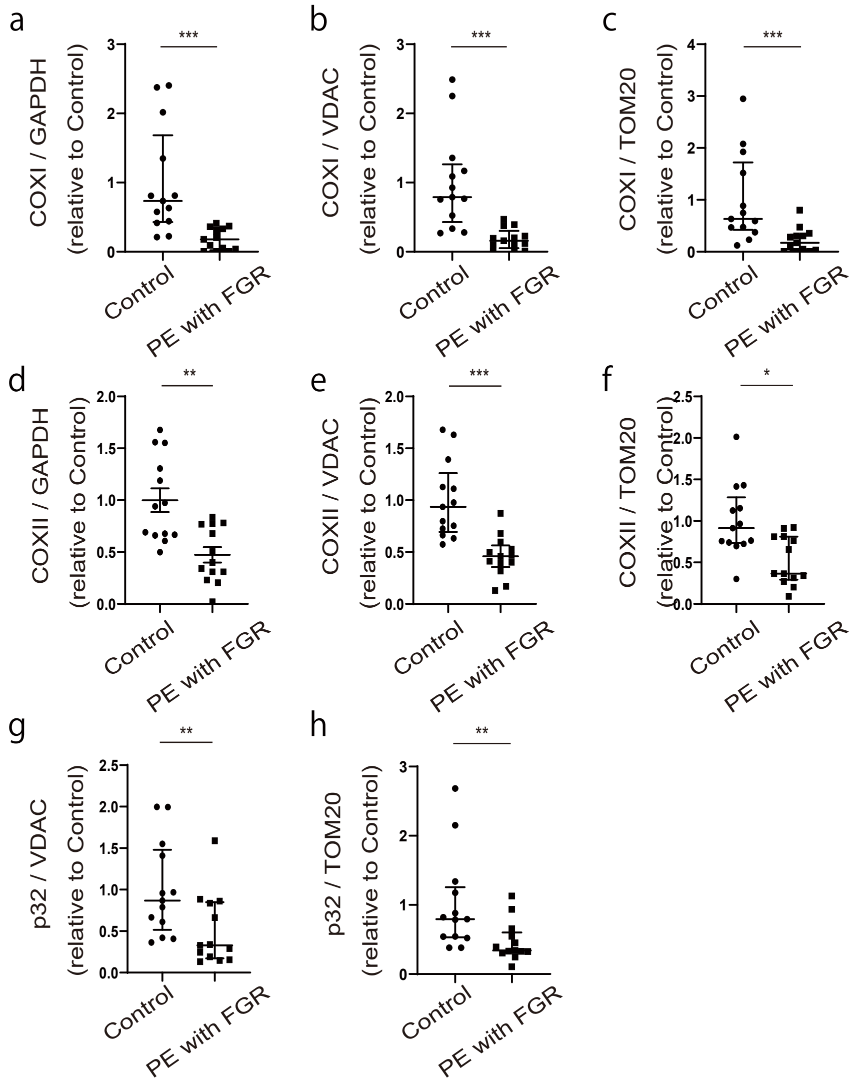

Supplementary Fig. 3 (**a**) Western blot quantification of placental COXI. GAPDH was used as an internal control. (**b**, **c**) Western blot quantification of placental COXI/VDAC and COXI/TOM20. COXI/VDAC and COXI/TOM20 indicate the amount of COXI per mitochondria. (**d**) Western blot quantification of COXII. GAPDH was used as an internal control. (**e**, **f**) Western blot quantification of placental COXII/VDAC and COXII/TOM20. COXII/VDAC and COXII/TOM20 indicate the amount of COXII per mitochondria. (**g**, **h**) Western blot quantification of placental p32/VDAC and p32/TOM20. p32/VDAC and p32/TOM20 indicate the amount of p32 per mitochondria. (**a**-**h**) Values are presented as the median with an interquartile range. Control, n=13; preeclampsia (PE) with FGR, n=13. The Mann–Whitney test was performed on control vs PE with FGR. *p<0.05, **p<0.01, ***p<0.001.

Supplementary Fig. 4

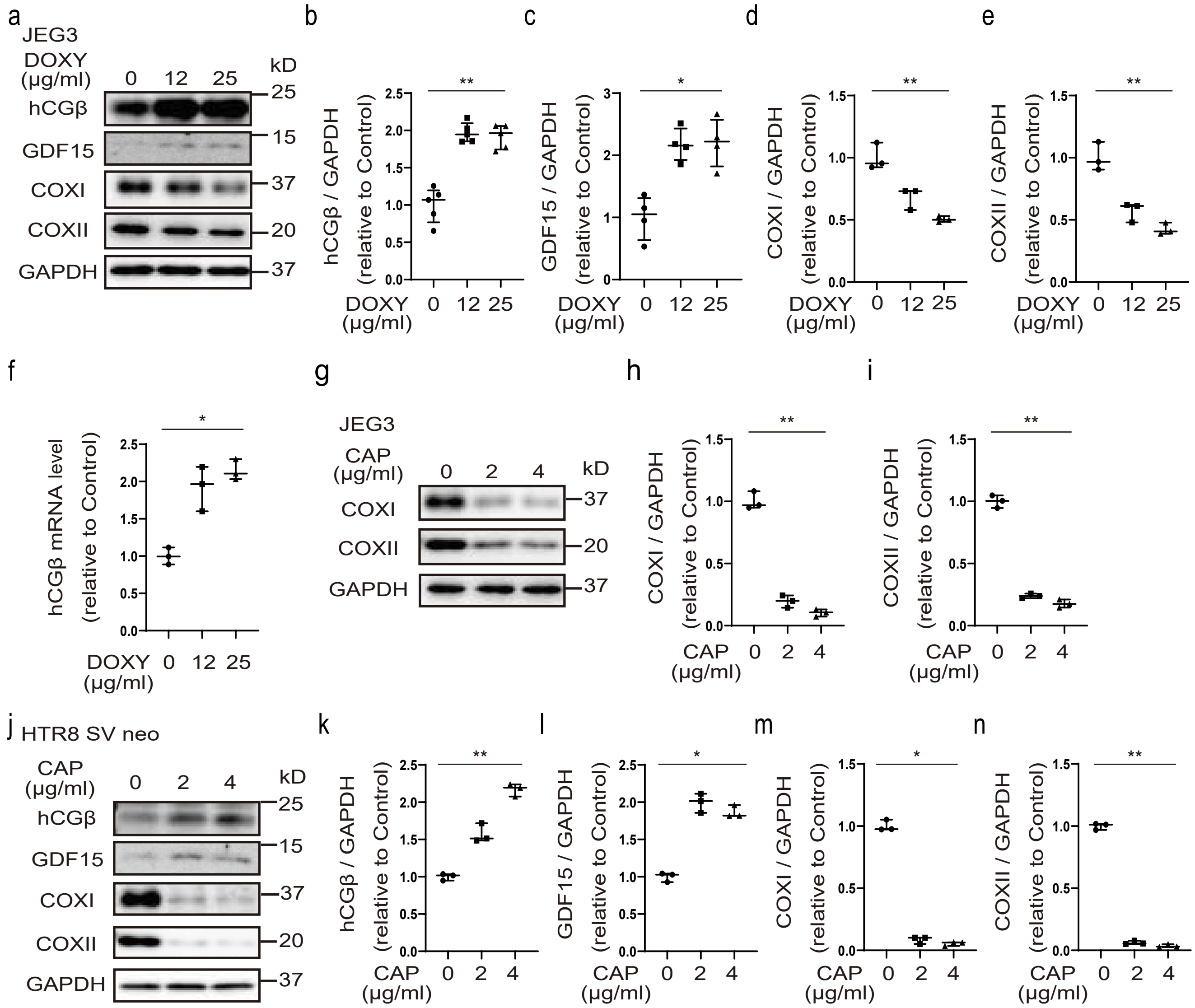

Supplementary Fig. 4 (**a**) Immunoblot analysis of human chorionic gonadotropin-beta (hCGβ), and growth differentiation factor 15 (GDF15), COXI, and COXII in JEG3 cells after doxycycline (DOXY) treatment for 72 h. GAPDH was used as an internal control. (**b**–**e**) Western blot quantification of hCGβ, GDF15, COXI, and COXII. (**f**) hCGβ mRNA expression in JEG3 cells after DOXY treatment for 72 h. (**g**) Immunoblot analysis of COXI and COXII in JEG3 cells after chloramphenicol (CAP) treatment for 72 h. GAPDH was used as an internal control. (**h, i**) Western blot quantification of COXI and COXII.
(**j**) Immunoblot analysis of hCGβ, GDF15,COXI, and COXII in HTR8 SV neo cells after CAP treatment for 72 h. GAPDH was used as an internal control. (**k**–**n**) Western blot quantification of hCGβ, GDF15, COXI, and COXII. (**b**-**f**, **h**, **i**, **k**-**n**) Values are presented as the median with an interquartile range of three, four, or five independent experiments. Statistical significance was assessed by the Kruskal–Wallis test. *p<0.05, **p<0.01. (**a**, **g, j**) The full unedited gels were shown in Supplementary information (Full unedited gel for Supplementary Fig.4).

Supplementary Fig. 5

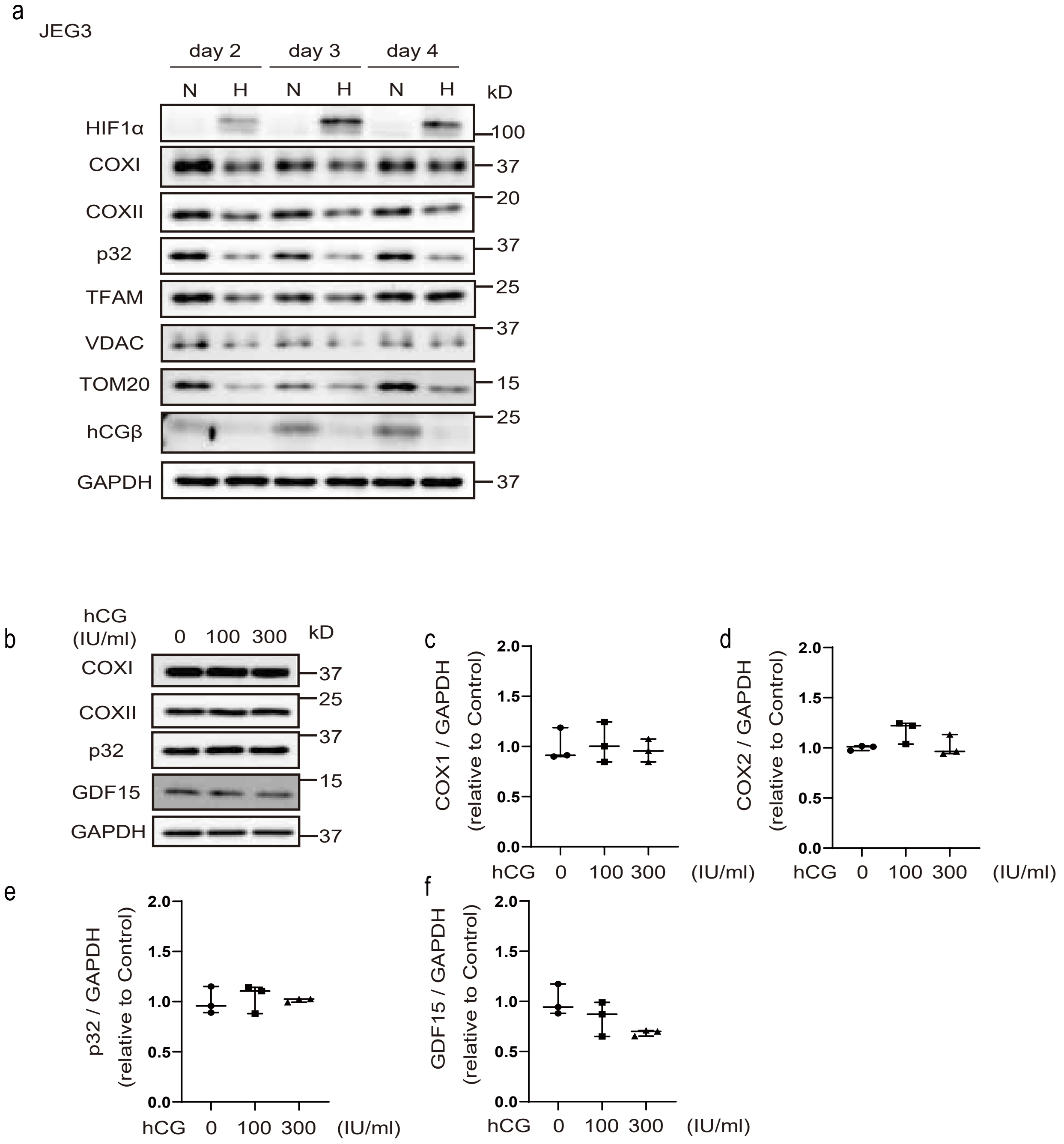

Supplementary Fig. 5 (**a**) Immunoblot analysis of HIF1α, COX1, COX2, p32, TFAM, VDAC, TOM20, and human chorionic gonadotropin-beta (hCGβ) in JEG3 cells under the normxic (N) (air,CO_2 5%) or hypoxic (H) (O_2 1%, CO_2 5%, NO_2 94%) conditions for 2, 3, and 4 days. GAPDH was used as an internal control.
(**b**) Immunoblot analysis of COXI, COXII, p32, and growth differentiation factor 15 (GDF15) in JEG3 cells after hCG treatment for 48 h.
(**c-f**) Western blot quantification of COXI, COXII, p32, and GDF15. GAPDH was used as an internal control. Values are presented as the median with an interquartile range of three independent experiments. Statistical significance was assessed by the Kruskal–Wallis test.
(**a**, **b**) The full unedited gels were shown in Supplementary information (Full unedited gel for Supplementary Fig.5).

Full unedited gel for Fig. 1

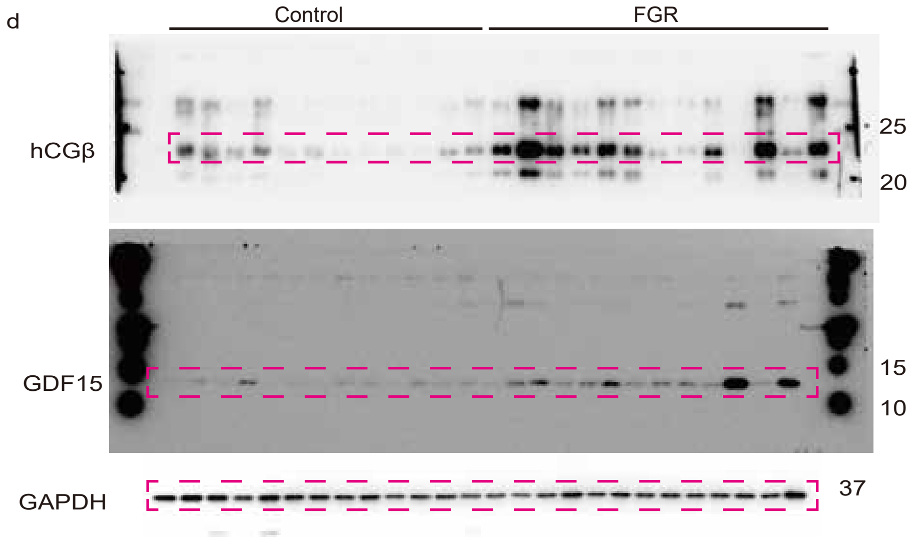

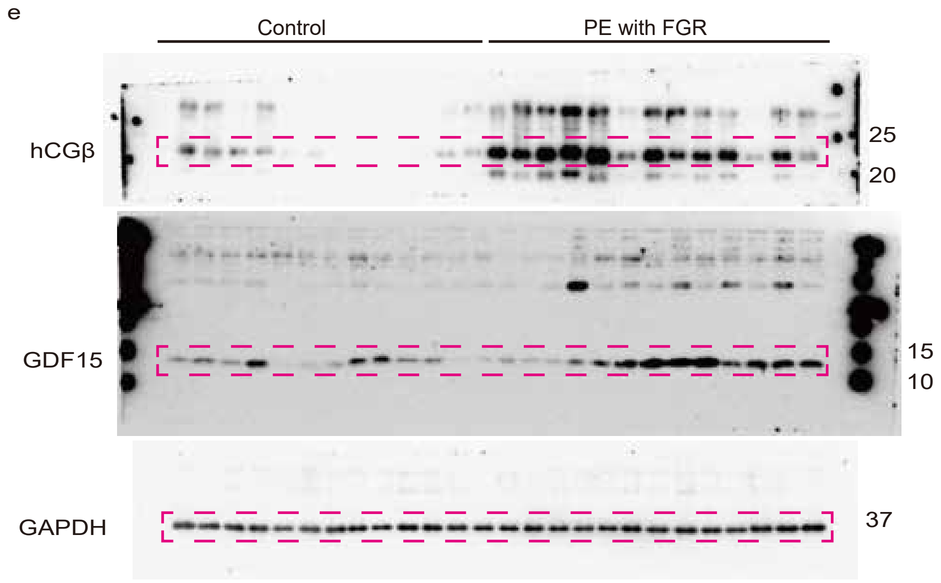

Full unedited gel for Fig. 3

e

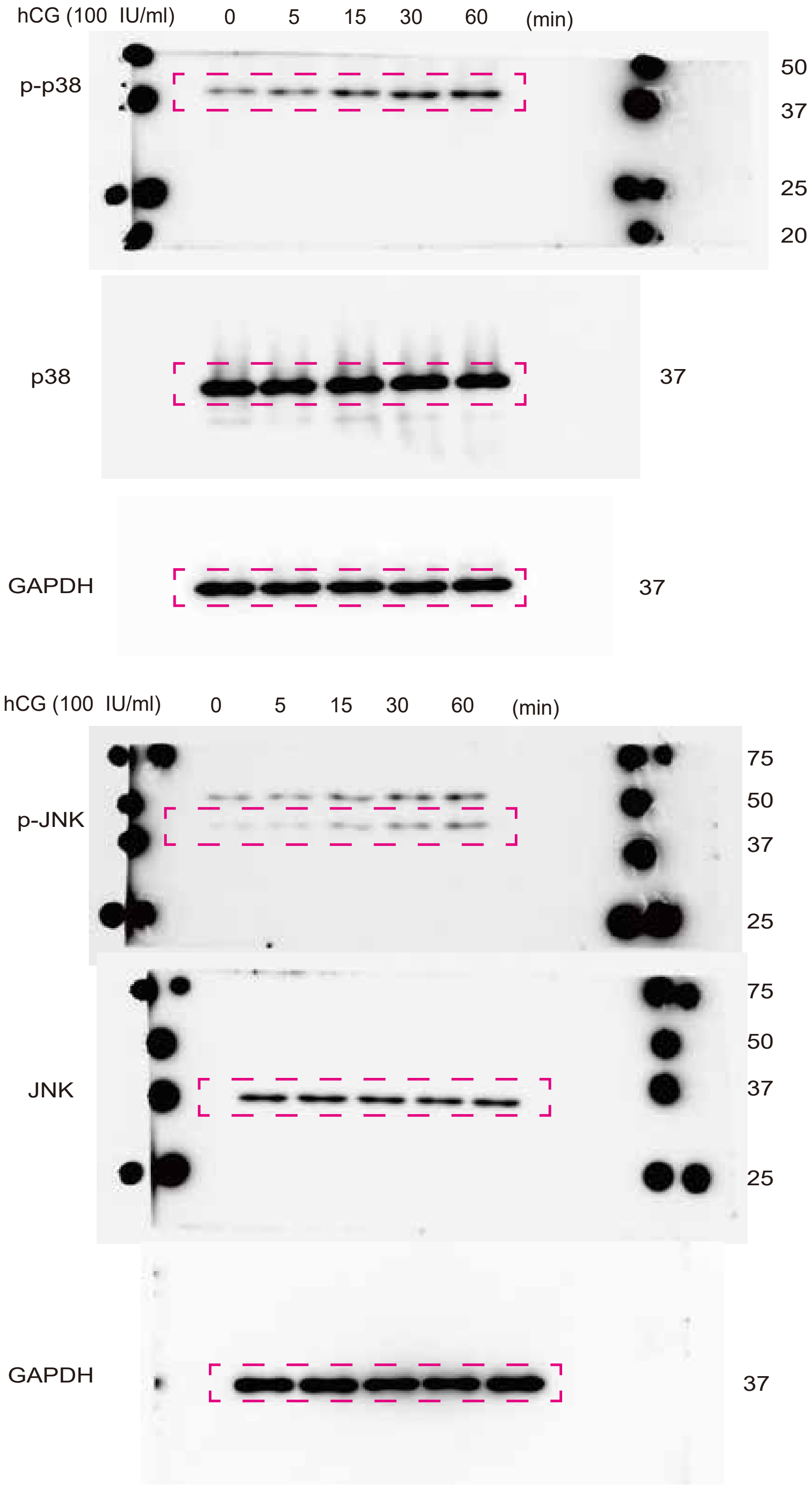

Full unedited gel for Fig. 4

a

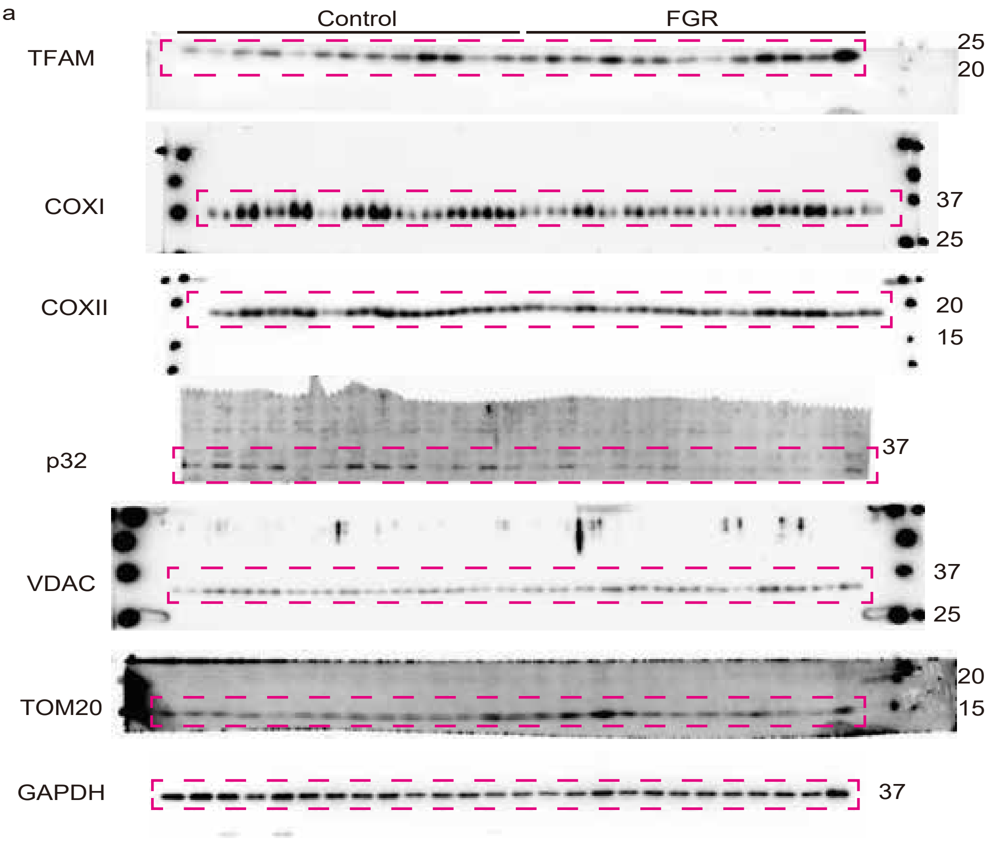

b

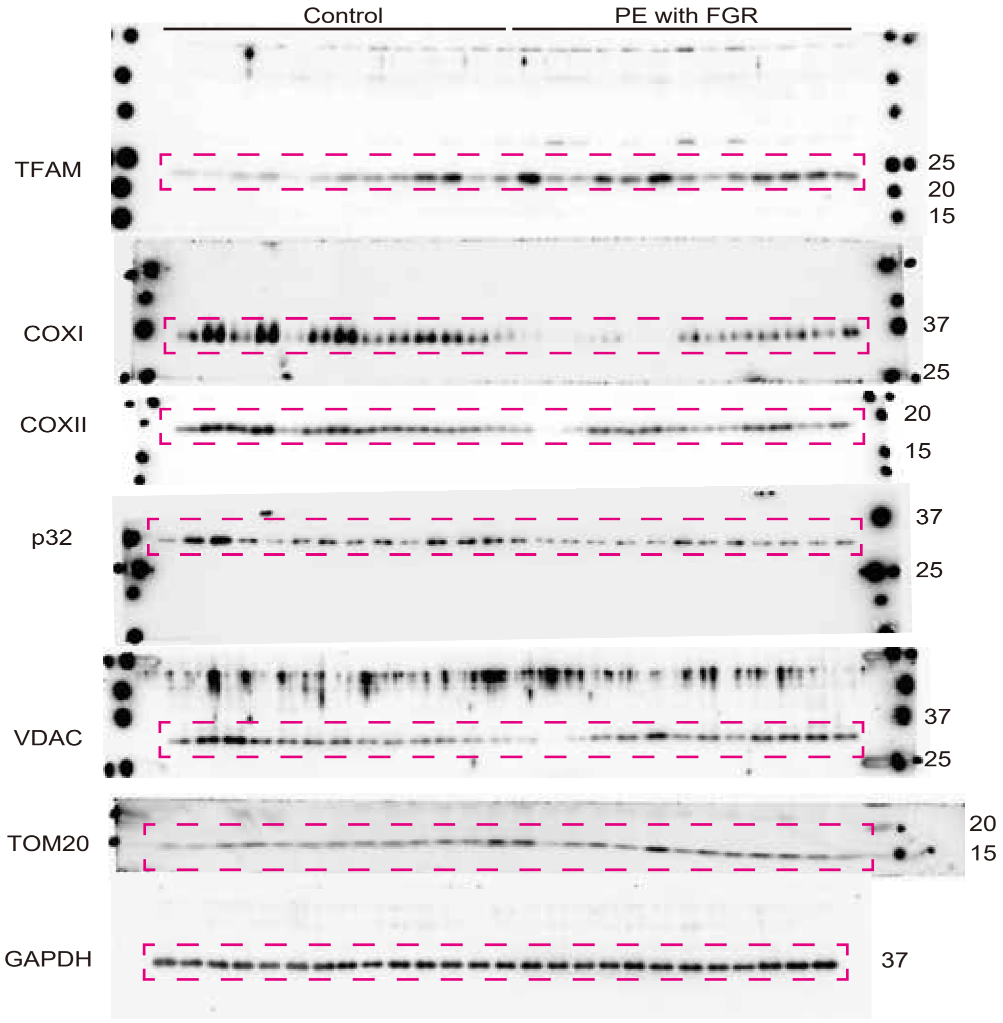

Full unedited gel for Fig. 5

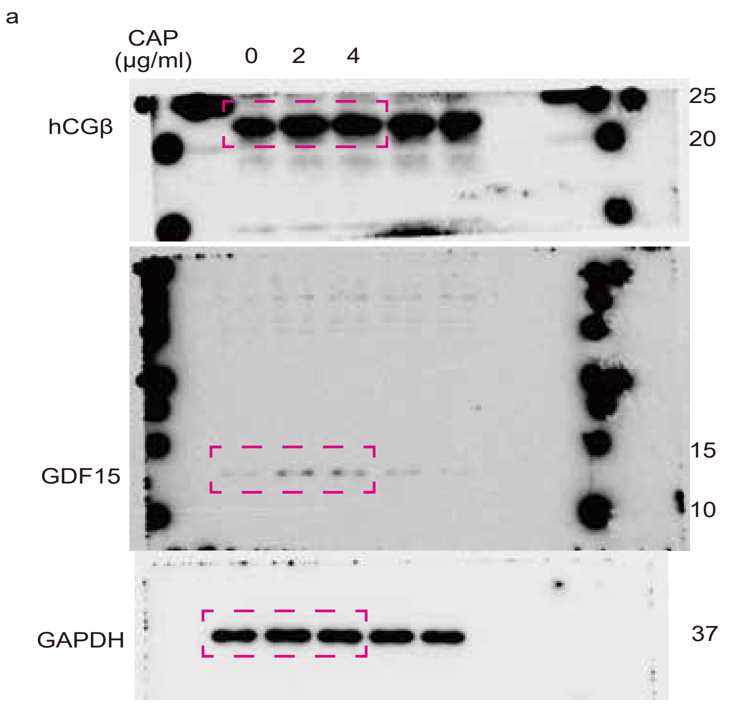

Full unedited gel for Fig. 6

a

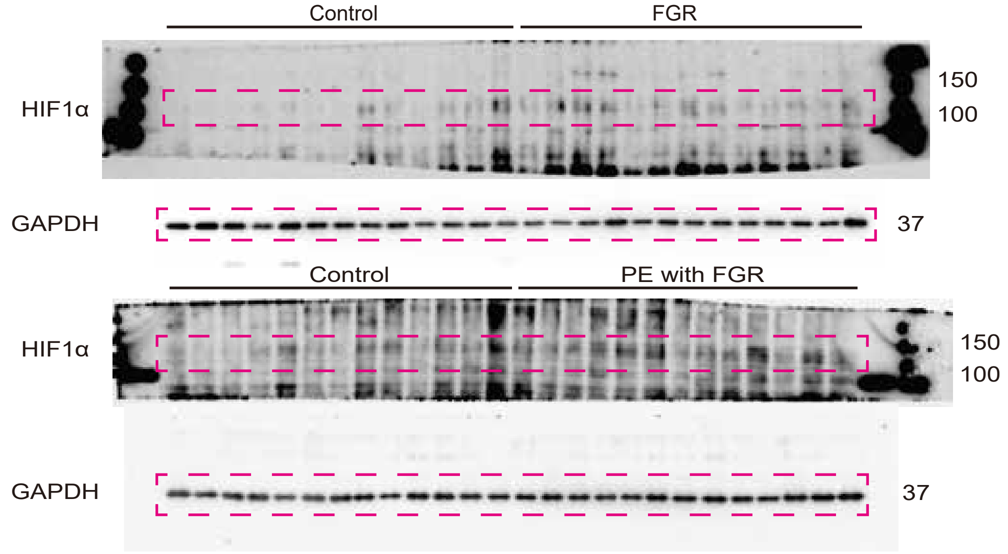

c

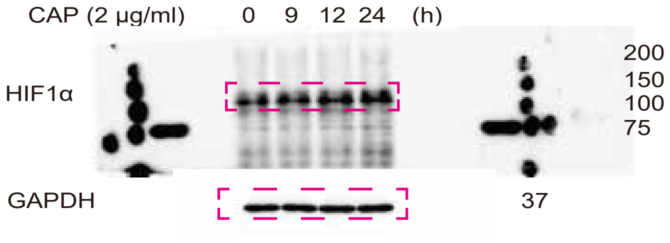

d

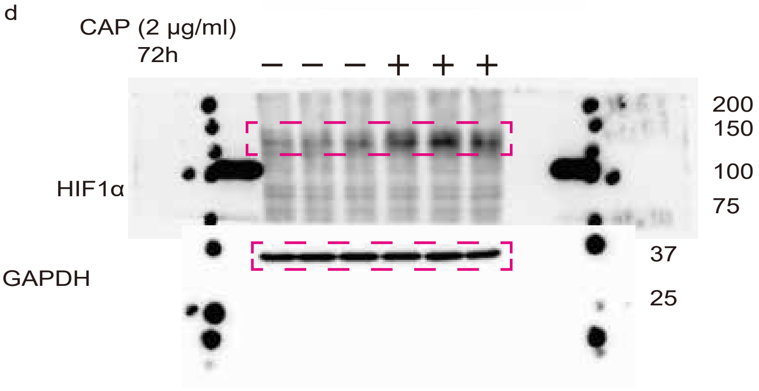

g

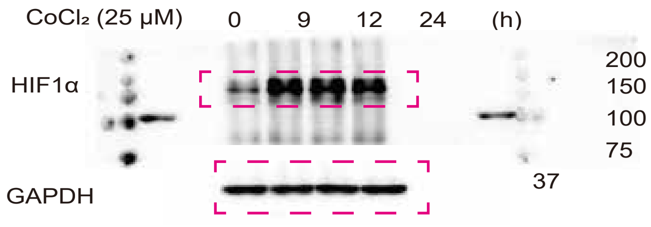

i

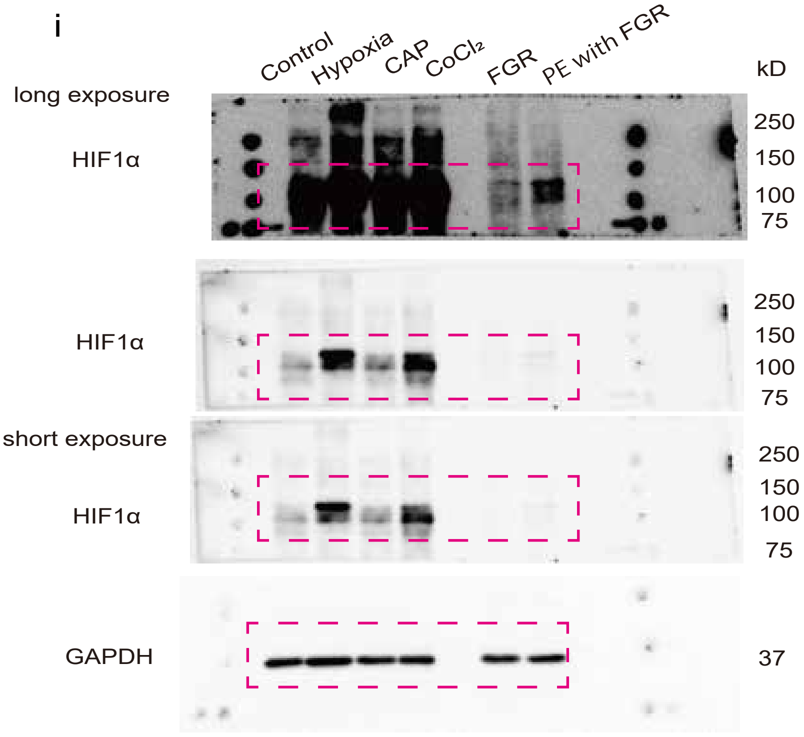

Full unedited gel for Supplementary Fig. 1

d

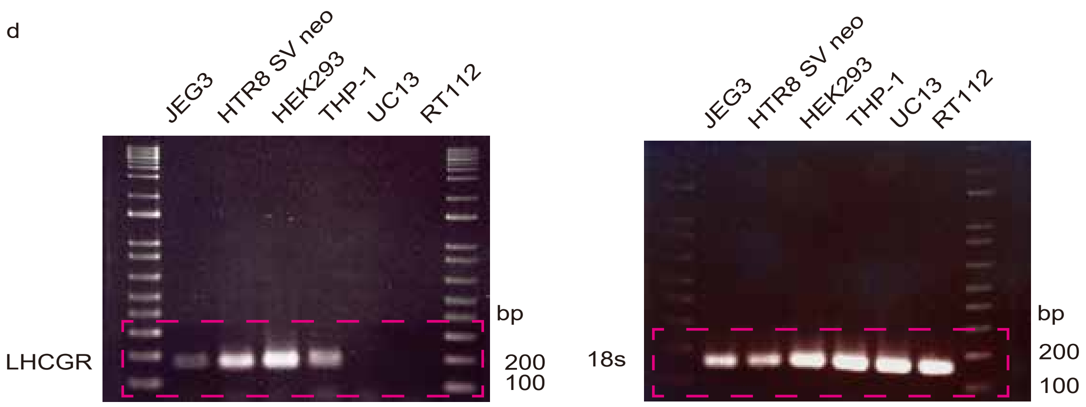

g

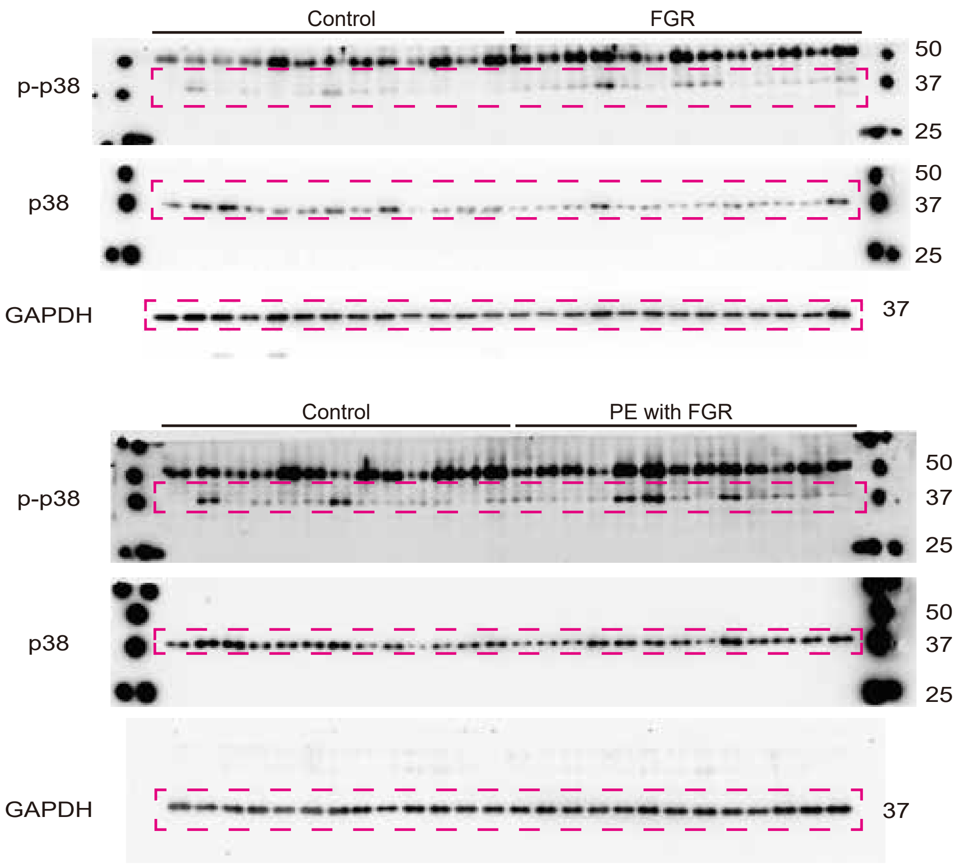

Full unedited gel for Supplementary Fig. 4

a

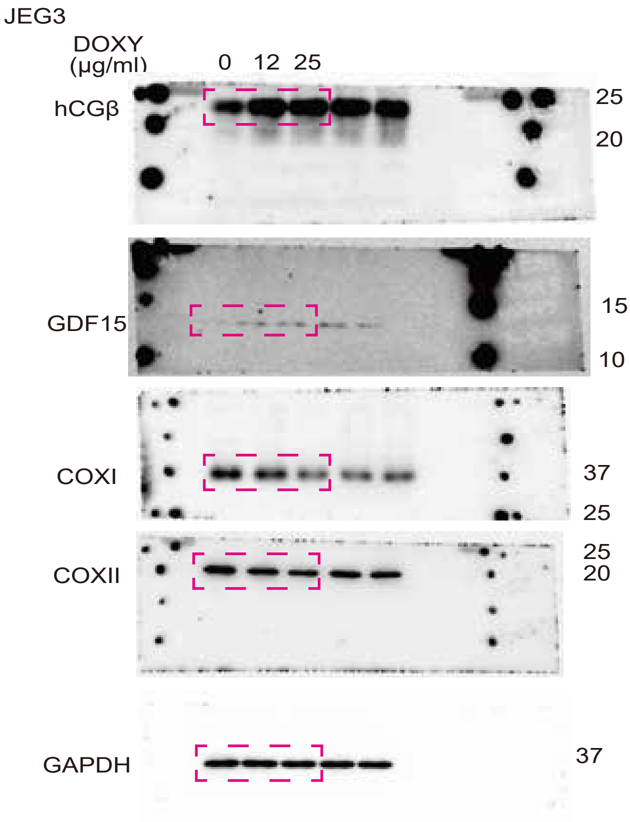

j

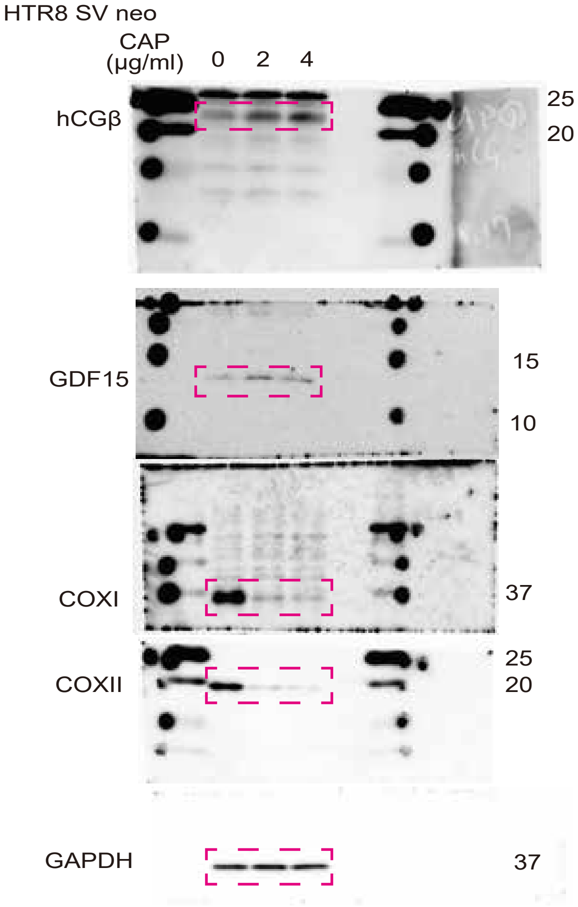

g

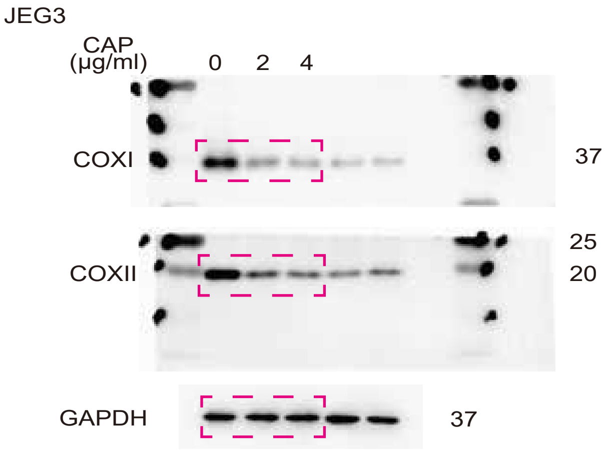

Full unedited gel for Supplementary Fig. 5

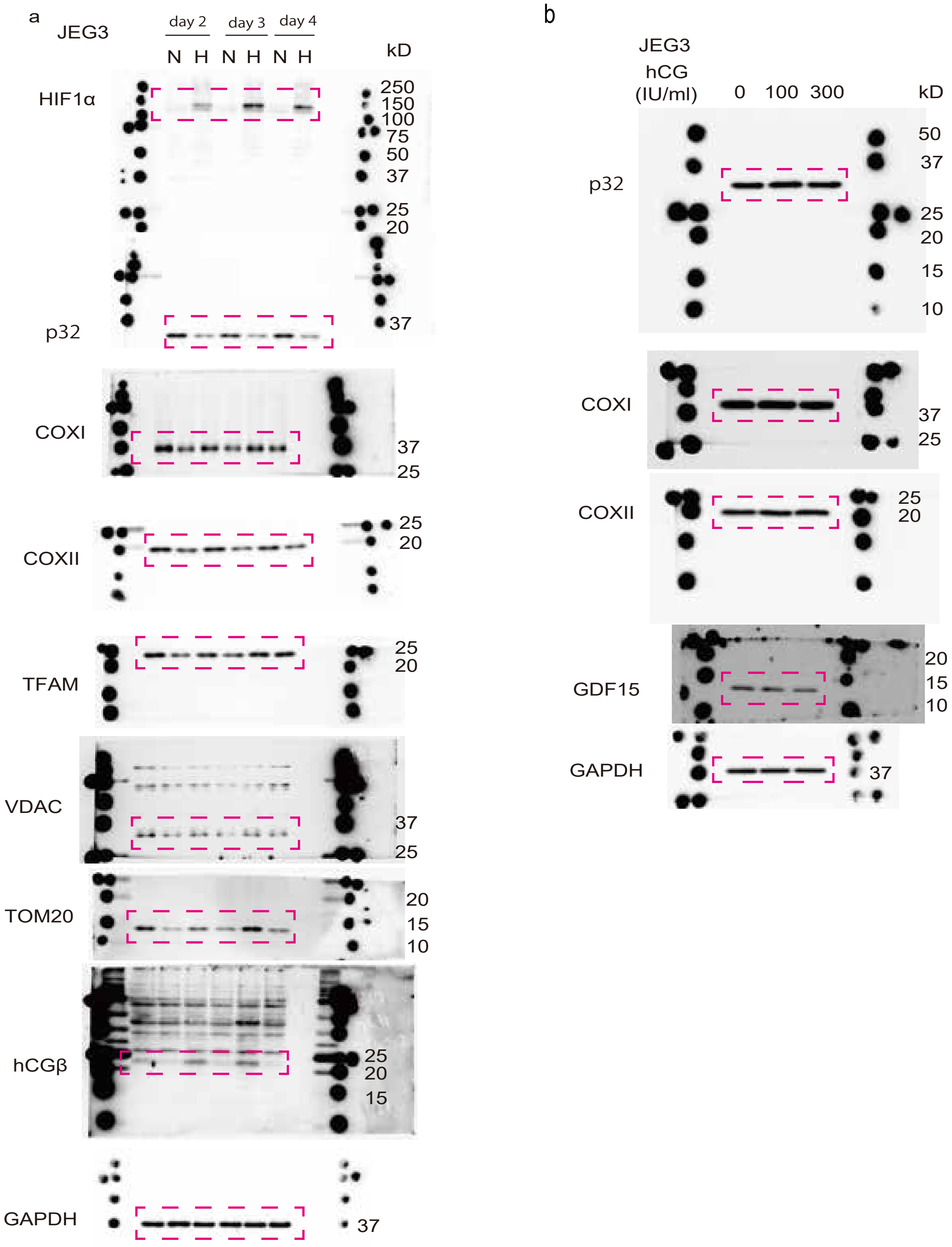

Supplement: Supplementary file 1 — Supplementary Information. [file 41598_2022_7893_MOESM1_ESM.pdf]
